# Cognitive effects of individual anticholinergic drugs: a systematic review and meta-analysis

**DOI:** 10.1590/1980-5764-DN-2022-0053

**Published:** 2023-05-29

**Authors:** Amirreza Naseri, Saeed Sadigh-Eteghad, Sepideh Seyedi-Sahebari, Mohammad-Salar Hosseini, Sakineh Hajebrahimi, Hanieh Salehi-Pourmehr

**Affiliations:** 1 Tabriz University of Medical Sciences, Student Research Committee, Tabriz, Iran.; 2 Tabriz University of Medical Sciences, Neurosciences Research Center, Tabriz, Iran.; 3 Tabriz University of Medical Sciences, Research Center for Evidence-Based Medicine, Iranian EBM Center: A Joanna Briggs Institute Center of Excellence, Tabriz, Iran.

**Keywords:** Cholinergic Antagonists, Cognition, Memory, Attention, Executive Function, Systematic Review, Meta-Analysis, Antagonistas Colinérgicos, Cognição, Memória, Atenção, Função Executiva, Revisão Sistemática, Metanálise

## Abstract

Anticholinergics (ACs) are among the most prescribed drugs. Investigating the impaired cognitive domains due to individual ACs usage is associated with controversial findings.

**Objective:**

The objective of this study was to investigate the effects of individual ACs on different aspects of cognitive function based on clinical trial studies.

**Methods:**

This systematic review was conducted following the PRISMA statement. A systematic search was performed in Embase, PubMed, Cochrane Library, Scopus, and Web of Science databases. Risk of bias (RoB) was assessed by the Joanna Briggs Institute checklists and the meta-analysis was performed using the CMA software.

**Results:**

Out of 3,026 results of searching, 138 studies were included. A total of 38 studies that assess the cognitive impacts of scopolamine were included in the meta-analysis. Included studies reported cognitive effects of scopolamine, mecamylamine, atropine, biperiden, oxybutynin, trihexyphenidyl, benzhexol, and dicyclomine; however, glycopyrrolate, trospium, tolterodine, darifenacin, fesoterodine, tiotropium, and ipratropium were not associated with cognitive decline. Based on the meta-analyses, scopolamine was associated with reduced recognition (SDM -1.84; 95%CI -2.48 to -1.21; p<0.01), immediate recall (SDM -1.82; 95%CI -2.35 to -1.30; p<0.01), matching to sample (SDM -1.76; 95%CI -2.57 to -0.96; p<0.01), delayed recall (SDM -1.54; 95%CI -1.97 to -1.10; p<0.01), complex memory tasks (SDM -1.31; 95%CI -1.78 to -0.84; p<0.01), free recall (SDM -1.18; 95%CI -1.63 to -0.73; p<0.01), cognitive function (SDM -0.95; 95%CI -1.46 to -0.44; p<0.01), attention (SDM -0.85; 95%CI -1.38 to -0.33; p<0.01), and digit span (SDM -0.65; 95%CI -1.21 to -0.10; p=0.02). There was a high RoB in our included study, especially in terms of dealing with possible cofounders.

**Conclusion:**

The limitations of this study suggest a need for more well-designed studies with a longer duration of follow-up on this topic to reach more reliable evidence.

## RESUMO.

Os anticolinérgicos (ACs) estão entre os medicamentos mais prescritos. Investigar os domínios cognitivos prejudicados devido ao uso individual de ACs está associado a achados controversos.

**Objetivo:**

Investigar os efeitos de ACs individuais em diferentes aspectos da função cognitiva, com base em estudos de ensaios clínicos.

**Métodos:**

Esta revisão sistemática foi realizada em acordo com a declaração PRISMA. Uma busca sistemática foi realizada nos bancos de dados Embase, PubMed, *Cochrane Library*, *Scopus* e *Web of Science*. O risco de viés (*risk of bias* - RoB) foi avaliado pelas listas de verificação do *Joanna Briggs Institute* e a meta-análise foi realizada através do *software* CMA.

**Resultados:**

Foram incluídos 138 estudos dos 3.026 resultados da pesquisa. Trinta e oito estudos que avaliam os impactos cognitivos da escopolamina foram incluídos na meta-análise. Os estudos incluídos relataram efeitos cognitivos de escopolamina, mecamilamina, atropina, biperideno, oxibutinina, triexifenidil, benzhexol, diciclomina; no entanto, glicopirrolato, tróspio, tolterodina, darifenacina, fesoterodina, tiotrópio e ipratrópio não foram associados ao declínio cognitivo. Com base nas meta-análises, a escopolamina foi associada a reconhecimento reduzido (DPM -1,84; IC95% -2,48 a -1,21; p<0,01), recordação imediata (DPM -1,82; IC95% -2,35 a -1,30; p<0,01), correspondência com a amostra (DPM -1,76; IC95% -2,57 a -0,96; p<0,01), recordação atrasada (DPM -1,54; IC95% -1,97 a -1,10; p <0,01), tarefas de memória complexas (DPM -1,31; IC95% -1,78 a -0,84; p<0,01), recordação livre (DPM -1,18; IC95% -1,63 a -0,73; p<0,01), função cognitiva (DPM -0,95; IC95% -1,46 a -0,44; p<0,01), atenção (DPM -0,85; IC95% -1,38 a -0,33; p<0,01) e amplitude de memória de dígitos (DPM -0,65; IC95% -1,21 a -0,10; p=0,02). Houve um alto RoB em nosso estudo, especialmente quanto aos possíveis confundidores.

**Conclusão:**

As limitações deste estudo sugerem a necessidade de estudos mais bem delineados e com maior duração de acompanhamento sobre o tema para alcançar evidências mais confiáveis.

## INTRODUCTION

Anticholinergics (ACs) are one of the most prescribed drug groups, with a wide variety of indications. Two groups of cholinoceptor antagonists include the muscarinic receptor antagonists, antimuscarinics, and nicotinic receptor antagonists, antinicotinics. Atropine, scopolamine, glycopyrrolate, tiotropium, and benztropine are examples of antimuscarinic drugs and mecamylamine is one of the antinicotinics. The uses of ACs range from disorders of the central nervous system (CNS) such as Parkinson's disease (PD) and motion sickness to ophthalmologic disorders, chronic obstructive pulmonary disease (COPD), cardiovascular disorders, gastrointestinal disorders like peptic ulcer disease, and finally lower urinary tract symptoms (LUTS)^1–3^. Patients with psychiatric problems are also the other users of these medications^4^. Antimuscarinic drugs can also be used as current standard care for laparoscopic surgery for a neuromuscular block in operating rooms^5^.

Cognition is a range of mental processes that include memory, executive function, attention, psychomotor speed, and social cognition. The effects of AC medication use on cognitive function is not a new field of interest in clinical research^6,7^, but considering the global aging and increasing chance of prescription of ACs^8^, it draws attention again in these years^9,10^. Blocking the action of acetylcholine, as one of the neurotransmitters involved in human cognition^11^, leads to cognitive side effects of ACs.

Numerous studies assess the possible relationship between ACs use and the risk of dementia^12^. Cognitive dysfunction due to the application of ACs could be one of the important factors associated with the impairment of quality of life related to AC burden^13,14^. A recent systematic review of 16 studies found an association between AC drug burden and delirium^15^. Also, another systematic review of 26 observational studies found an association between any AC usage and the incidence of dementia and cognitive decline but not the incidence of mild cognitive impairment (MCI)^16^. Also, another meta-analysis found that the usage of ACs for ≥3 months is associated with an increased risk of dementia^17^.

Impairment in cholinergic neurotransmission is associated with the progression of Alzheimer's disease (AD), delirium, and medication-induced cognitive impairment^18^. Investigating the impaired cognitive domains due to AC usage is associated with controversial findings. A recent observational study over 4 years found an impairment in the speed of processing as the only cognitive domain associated with AC usage^19^. Great diversity in cognitive outcomes based on different cognitive assessments^20^ recommended a domain-specific role of ACs on human cognitive function.

This systematic review aimed to assess the effects of each AC drug on cognitive function in individuals without neuropsychiatric disease. Also, as the secondary outcome, in the meta-analysis, this study assesses the impact rate of scopolamine on each cognitive domain in healthy young people.

## METHODS

This systematic review is conducted following the Preferred Reporting Items for Systematic Reviews and Meta-Analyses (PRISMA) statement^21^.

### Inclusion and exclusion criteria

The population, interventions, comparisons, outcomes, and study designs (PICOS) of this systematic review are as follows:

- Population: cognitively intact individuals without any neurological or psychological disorders at any age (without any age limitation);
- Intervention: AC drugs in any dosage and route of administration;
- Comparison: placebo or control group without using any centrally active drug;
- Outcome: cognitive function, memory, attention, psychomotor speed, or executive function, based on any assessments;
- Type of studies: randomized or non-randomized clinical trials.

Studies with the abovementioned criteria were included in the systematic review. Studies of patients with neurological as well as psychiatric disorders were excluded. Animal studies, conference abstracts, and other types of articles, including observational studies, baseline-controlled trials, reviews, case reports, letters, and comments, studies in any languages except English, withdrawn studies, and finally studies without access to full text were also excluded from the systematic review.

For the meta-analysis, only studies in which the cognitive effects of a single scopolamine administration, in any dose, on healthy young individuals, with any route of administration were compared with the control group were included. Studies of patients with non-neuropsychiatric diseases (e.g., LUTS, COPD, and overactive bladder (OAB)), as well as studies on the elderly population or children were excluded from the meta-analysis.

### Search methods for identification of studies

An electronic search in Embase, PubMed, Cochrane Library, Scopus, and Web of Science databases was conducted in February 2020 and updated in February 2021. The detail of searching keywords is presented in Supplementary Material 1. For full coverage of published studies, the reference lists and citations of each included study as well as the review articles in this field were also checked.

### Study selection

Two independent researchers assess the eligibility of the search results in two title/abstract and full-text stages. In the case of disagreements, the authors resolved them by discussion, and if a consensus was not reached, the other author, who is an expert in this field, helped them to resolve it.

### Data extraction

Two researchers extracted the data using a table. Data include the name of the first author, publication year, type of the study, the condition of the participants, sample size, mean age, number of male participants, the cognitive test name, the interval between medication usage and cognitive assessment, the type and consumption of AC medicine, route of administration, and the results of cognitive assessments. Any disagreement was resolved in consultation with the third author.

### Risk of bias assessment

The risk of bias (RoB) assessment in included studies was conducted by the Joanna Briggs Institute (JBI) checklists^22^. Two researchers assess the RoB and in case of any problem, the third person resolved it.

### Data synthesis and analysis

Meta-analysis was performed using the second version of comprehensive meta-analysis (CMA.2) software. The numerical values after medicine or placebo usage in types of mean and standard deviation (SD), mean and standard error (SE), and mean and 95% confidence intervals were converted into a single effect size and imported into the final analysis. The final analysis was conducted with a 95% confidence interval (CI) and 0.05 level of significance for p-value and reported in the form of standard difference in means (SDM). The heterogeneity among the results was assessed using the I^2^ index and in case of significant heterogeneity (I^2^>50% and p-value<0.05), a random-effect model was used for meta-analysis. Also, Begg and Mazumdar's correlation was used for assessing the publication bias. The final results are presented in forest and funnel plots.

## RESULTS

### Results of the search

**Figure 1:**
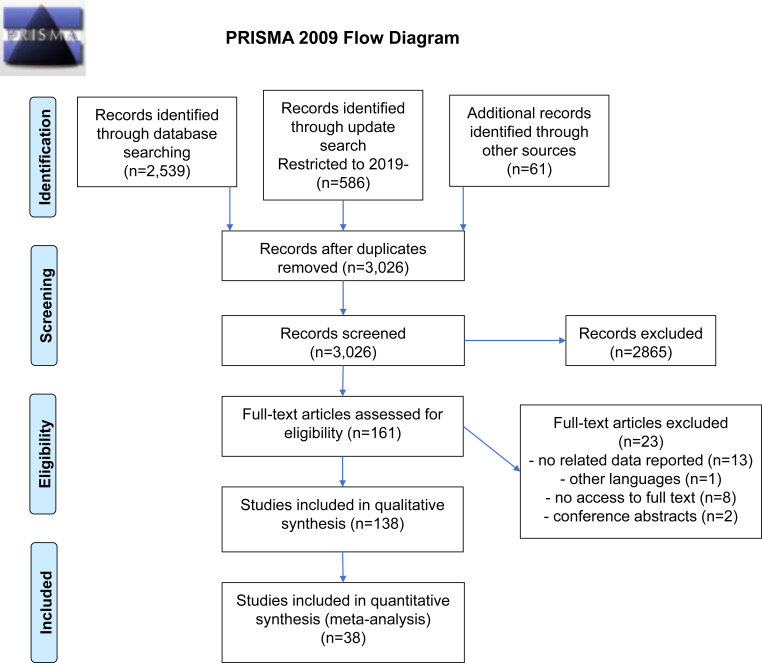
PRISMA flow diagram.

Finally, out of 3,026 results of the electronic searches and hand searching, 138 studies met our eligibility criteria. The details of the screening process are presented in the PRISMA flow diagram (Figure 1). Out of these 138 studies, 38 studies that assessed the cognitive impact of a single administration of scopolamine in any dosage in healthy young volunteers were included in the quantitative synthesis.

### Characteristics of included studies and summary of findings

Supplementary Material 2 shows the detailed characteristics of the included studies and a summary of findings. A total of 138 studies including 75 RCTs and 63 quasi-experimental studies assess the effects of different doses of scopolamine, mecamylamine, glycopyrrolate, atropine, biperiden, trospium, tolterodine, oxybutynin, darifenacin, dicyclomine, fesoterodine, tiotropium, trihexyphenidyl, benzhexol, and ipratropium. Different routes of administration, including intramuscular, intravenous, and subcutaneous injection, infusing, oral, intranasal, or transdermal, were used in the studies. Also, a wide range of cognitive tests was used in the studies, which include recall tasks, recognition tasks, reaction time assessments, vigilance tasks, learning tasks, and a higher level of mental processing tasks including judgment and reasoning tasks. The participants of the studies were healthy volunteers in most of the articles. Benign leiomyoma uteri (one study), urinary incontinence (three studies), surgical candidates (three studies), OAB (three studies), and COPD (two studies) were the other baseline conditions. Except for 27 studies in older adults and 2 studies in children, the rest of the studies were conducted on adult participants.

### Scopolamine

**Figure 2:**
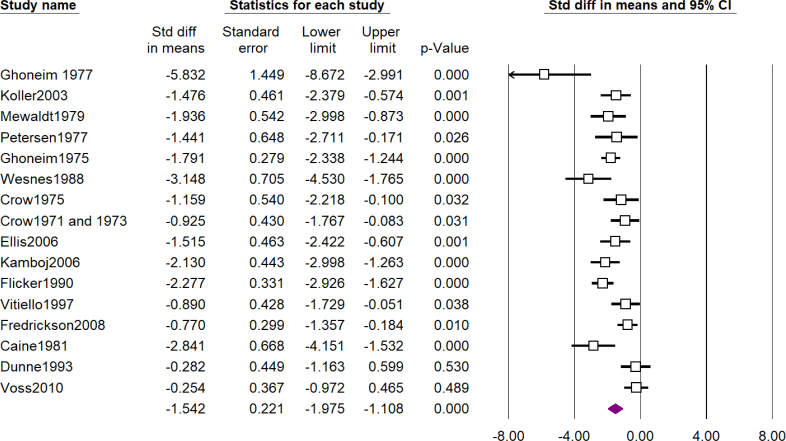
Forest plot of assessment of effects of scopolamine on delayed recall (see Supplementary Material 2).

**Figure 3:**
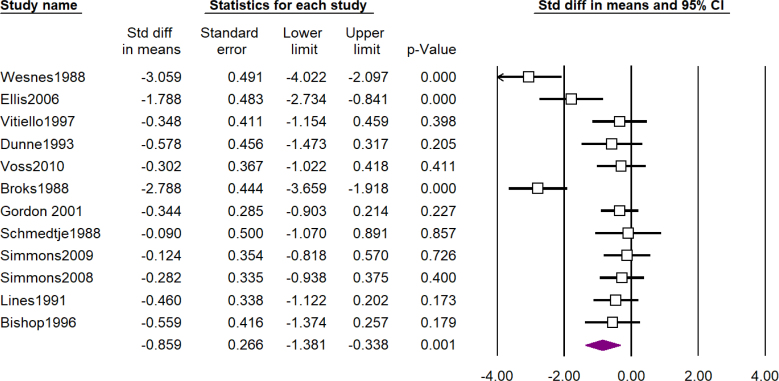
Forest plot of assessment of effects of scopolamine on attention (see Supplementary Material 2).

**Figure 4:**
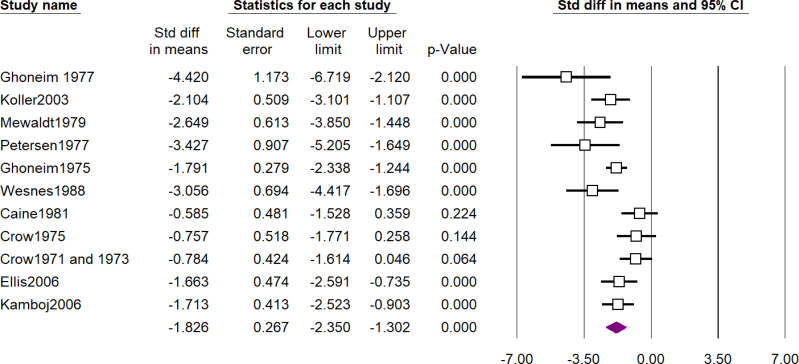
Forest plot of assessment of effects of scopolamine on immediate recall (see Supplementary Material 2).

A total of 101 studies assessed the cognitive effects of scopolamine and 3 of them assessed the effects of methscopolamine as well. Table 1 shows a summary of the results of meta-analyses and forest plots are presented in Figures 2, 3, and 4 and Supplementary Material 3. The most impressive effect of scopolamine was in terms of recognition (SDM -1.84; 95%CI -2.48 to -1.21; p<0.01), immediate recall (SDM -1.82; 95%CI -2.35 to -1.30; p<0.01), and matching to sample tasks (SDM -1.76; 95%CI -2.57 to -0.96; p<0.01), followed by delayed recall (SDM -1.54; 95%CI -1.97 to -1.10; p<0.01), complex memory tasks (SDM -1.31; 95%CI -1.78 to -0.84; p<0.01), free recall (SDM -1.18; 95%CI -1.63 to -0.73; p<0.01), cognitive function (SDM -0.95; 95%CI -1.46 to -0.44; p<0.01), and attention (SDM -0.85; 95%CI -1.38 to -0.33; p<0.01), and finally digit span was the least impaired task (SDM -0.65; 95%CI -1.21 to -0.10; p=0.02); nevertheless, the difference between scopolamine and placebo was significant in all of the investigated outcomes.

**Table 1:** Summary of results of meta-analysis.

Outcome	Number of studies	Heterogeneity		Meta-analysis model	Standard difference in means	Standard error	95% confidence intervals		p-value
		I^2^ (%)	p-value				Lower limit	Upper limit	
Attention	12	81.69	<0.01*	Random effect	−0.85	0.26	−1.38	−0.33	<0.01*
Delayed recall	16	73.27	<0.01*	Random effect	−1.54	0.22	−1.97	−1.10	<0.01*
Digit span	6	72.80	<0.01*	Random effect	−0.65	0.28	−1.21	−0.10	0.02*
Free recall	7	53.53	0.04*	Random effect	−1.18	0.23	−1.63	−0.73	<0.01*
Immediate recall	11	65.85	<0.01*	Random effect	−1.82	0.26	−2.35	−1.30	<0.01*
Matching to sample	7	86.99	<0.01*	Random effect	−1.76	0.41	−2.57	−0.96	<0.01*
Cognitive function	14	84.59	<0.01*	Random effect	−0.95	0.26	−1.46	−0.44	<0.01*
Complex memory tasks	18	80.57	<0.01*	Random effect	−1.31	0.24	−1.78	−0.84	<0.01*
Recognition	13	84.17	<0.01*	Random effect	−1.84	0.32	−2.48	−1.21	<0.01*

* Note: Statistically significant.

### Mecamylamine

The cognitive effects of mecamylamine were investigated in 11 studies and impairment in adaptive tracking performance, alertness, learning tasks, continuous performance test, and inspection time was reported in 8 studies. In the rest of the three studies, the cognitive effect of mecamylamine was not significant.

### Scopolamine and mecamylamine

Based on the results of four studies, a combination of muscarinic and nicotinic receptor antagonism with both mecamylamine and scopolamine was associated with worse outcomes compared to each one, alone.

### Glycopyrrolate

As expected, glycopyrrolate as a peripherally active AC did not impose a cognitive deterioration in any of the three included studies.

### Atropine

Six studies assess the cognitive effects of atropine. The drug's negative effects on the Stroop test and Gottschaldt's Hidden Figure Test in one study, repeated acquisition in one study, and digit recall in one study were reported, while in three studies, administration of atropine was not associated with significant cognitive impairment.

### Biperiden

Five of the included studies assess the cognitive effects of biperiden. The drug usage was associated with impairment in episodic memory, visuospatial processes, motor learning, verbal learning task, continuous recognition memory test, spatial memory task, word retrieval task, and delayed recall task, but the drug did not impair sustained attention, n-back, and behavioral learned irrelevance index measures.

### Trospium

None of the five studies assessing the cognitive effects of trospium could detect a meaningful relation between using trospium and impairment in different cognitive tasks.

### Tolterodine

None of the five included studies could detect a meaningful difference between tolterodine and placebo usage in terms of cognitive function.

### Oxybutynin

Four studies found no effect of oxybutynin in cognitive function, while significant impairment was observed in Buschke selective reminding test and reaction time, memory performance, and other cognitive tests in the remaining three studies.

### Darifenacin

None of the four studies assessing the cognitive effects of darifenacin reported an associated significant impairment in cognitive function.

### Fesoterodine

The cognitive effects of fesoterodine were investigated in three different studies and there was not a statistically significant difference between the drug and placebo in none of the cognitive assessments in these studies.

### Trihexyphenidyl

Four studies of assessment of the cognitive function after using trihexyphenidyl met our inclusion criteria and usage of this drug was associated with impairment in delayed recall tasks in 2 studies, while other cognitive assessments were not significantly different between the trihexyphenidyl and control.

### Others

One study assessing the cognitive function after tiotropium usage did not report a significant impairment in cognitive function. The effects of benzhexol on cognitive function were assessed in one study and impairment in delayed recall, short story, and orientation test was reported in one study. The cognitive effects of ipratropium were assessed in one study in which drug usage was not associated with significant impairment. Only one study reported the cognitive effects of dicyclomine. In this study, using this drug was effective on cognitive function based on simple reaction time, working memory task, and picture recognition task.

### Risk of bias of the included studies

The details of the RoB assessment are presented in Supplementary Material 4. Except for 15 studies, in the rest of the RCTs, the method of randomization was not mentioned. Also, 29 RCTs and 40 non-randomized clinical trials did not clarify the detailed method of dealing with possible confounding factors, which could affect the results of the studies. Studies without a control group were excluded from our study. A lack of multiple measurements of the outcome was the other source of bias in quasi-experimental studies. Also, the funnel plot of studies is presented in Supplementary Material 4. After removing one study^23^, based on Begg and Mazumdar's correlation, we found no publication bias in the meta-analysis.

## DISCUSSION

As the main outcome, this study aimed to assess the effects of each AC drug on cognitive function. Our included studies reported that using glycopyrrolate, trospium, tolterodine, darifenacin, fesoterodine, tiotropium, and ipratropium was not associated with a significant decline in cognitive function; however, using scopolamine, mecamylamine, atropine, biperiden, oxybutynin, trihexyphenidyl, benzhexol, and dicyclomine seems to impair the cognitive ability based on clinical evidence.

From the mechanism point of view, ACs inhibit binding of the acetylcholine, a neurotransmitter, which has a crucial role in memory and cognitive function^24–26^. Acetylcholine receptors have two major subtypes, namely, muscarinic (e.g., M1–M5) and nicotinic receptors (e.g., α7, α4β2, and α3β4)^27–29^. Muscarinic M1 receptors, as the most predominant muscarinic receptor in memory and cognition, have the highest concentration in cortical regions, including the hippocampus^30,31^. M2 and M3 subtypes, which are considered the mainstay of treatment for LUTS, are expressed in high density in the heart and smooth muscles^32^. Using nonselective antimuscarinic drugs for indications like controlling the symptoms of LUTS is associated with cognitive worsening by blocking of M1 muscarinic receptor in the CNS. This can be more challenging in dementia patients because of the reduction of brain acetylcholine activity^33,34^. Regarding the nicotinic receptors, the involvement of nicotinic α7 receptors in working memory as well as the α4β2 subtype in attention have been reported. Also, modulation of depression and anxiety is another mechanism of nicotinic receptors’ involvement in cognition^35^.

Scopolamine, as an AC drug that blocks all subtypes of muscarinic cholinergic receptors, used as a model for cognitive dysfunction in animal and human studies for many years^36^. The results of our meta-analysis based on scopolamine prove the involvement of muscarinic receptors in almost all aspects of cognitive function. Atropine as one of the widely used ACs in surgery with a similar mechanism of action to scopolamine^37^ is associated with lesser cognitive effect, compared with scopolamine^38^, but our study demonstrated a significant cognitive effect of atropine, compared to placebo/control. Oxybutynin is a selective M1 and M3 (and M2) receptor antagonist^39^, which was associated with cognitive worsening in our included studies.

In children, oxybutynin is the most prescribed first-line therapy for OAB^40^. A recent systematic review found that using the AC in children is not associated with poor cognitive outcomes^41^. Only two of our included studies had children participants and, in these studies, using oral oxybutynin and tolterodine was not associated with cognitive impairment in children^42,43^. Reports of biperiden, an M1 receptor antagonist, induced delirium in children and adolescents^44,45^ and recommended more caution in using this drug in each age group.

Regarding the route of administration, transdermal scopolamine was not associated with significant cognitive effects in three out of five included studies. Despite the efficacy in reducing symptoms, multiple observational studies could not find a meaningful association between transdermal oxybutynin use and risk of cognitive impairment as well^46–49^. There are limited studies on the assessment of cognitive effects of mydriatic eye solutions, which mostly include ACs. A study based on Montreal's cognitive assessment could not detect a significant difference in cognitive function with and without using the mydriatic solutions^2^. None of our included studies assess the cognitive safety of AC eye drop solutions.

A systematic review of assessing the cognitive function in patients using the ACs, only based on MMSE, found that oxybutynin has the largest cognitive effect followed by darifenacin and tolterodine^50^. Although baseline-controlled studies reported some cognitive effects of tolterodine^51^, the results of our study are the same as the findings of this systematic review, so none of the studies on darifenacin and tolterodine users reported a cognitive impairment after using these drugs.

This study did not include all of the ACs. Reports of cognitive impairment after using imidafenacin, as a newly developed antimuscarinic drug, are limited to case reports^52^. Although imidafenacin is an antagonist of M1 and M3 receptors, *in vivo* studies found fewer brain muscarinic receptors occupation^53,54^. Also, the cognitive safety of this drug has been reported in numerous studies^55–58^, but no study met our inclusion criteria for this systematic review. Also, to the best of our knowledge, the cognitive safety of revefenacin, a long-acting muscarinic antagonist for the treatment of COPD, is not yet assessed in clinical trials^59^.

This study was associated with multiple limitations. The first one is diversity in ACs in different studies which prevents us to reach a comprehensive meta-analysis. Also, the heterogeneity in drug consumption was the other source of diversity. One of the strengths was including only the clinical trial studies and excluding the observational studies. We carefully extracted the data from each study and used a standard approach in conducting this review. Also, adding other resources to search results of databases led to the full coverage of published studies.

In conclusion, considering the limited number of well-designed RCTs, this systematic review found that glycopyrrolate, trospium, tolterodine, darifenacin, fesoterodine, tiotropium, and ipratropium are not associated with worsening of human cognition, but scopolamine, mecamylamine, atropine, biperiden, oxybutynin, trihexyphenidyl, benzhexol, and dicyclomine should be administered with caution. Also, the results of our meta-analysis indicate that the most impaired cognitive domain after using scopolamine, a nonselective muscarinic receptor antagonist, is recognition and immediate recall but in general, all aspects of human cognition are impaired by this drug. There is a need for more well-designed studies with a longer duration of follow-up to obtain better evidence in this regard.
