# Supplementary material for: Cognitive effects of individual anticholinergic drugs: a systematic review and meta-analysis
Source: Dement Neuropsychol. 2023 May 29;17:e20220053. doi: 10.1590/1980-5764-DN-2022-0053 (PMC10229087; doi:10.1590/1980-5764-DN-2022-0053)
Supplement: Supplementary file 1 [file 1980-5764-DN-17-e20220053-Suppl01.docx]

**Supplementary Material 1**

**Embase**

Session Results : Date: 29 Feb 2020

| No. | Query | Results |
| --- | --- | --- |
| #1. | 'anticholinergic'/exp | 235,832 |
| #2. | 'anticholinergic':ab,ti | 14,394 |
| #3. | 'cholinergic antagonist'/exp | 235,832 |
| #4 | 'cholinergic antagonist':ab,ti | 647 |
| #5. | cholinergic AND antagonist:ab,ti | 9,144 |
| #6. | 'muscarinic receptor blocking agent'/exp | 123,979 |
| #7. | antimuscarinic:ab,ti | 3,298 |
| #8. | muscarinic:ab,ti | 34,239 |
| #9. | #1 OR #2 OR #3 OR #4 OR #6 OR #7 OR #8 | 256,349 |
| #10. | 'mental state'/exp | 154,575 |
| #11. | mental AND state:ti,ab | 76,142 |
| #12. | 'cognition'/exp | 2,256,866 |
| #13. | cognit*:ab,ti | 527,393 |
| #14. | 'memory'/exp | 293,298 |
| #15. | 'memory':ab,ti | 311,034 |
| #16. | 'learning'/exp | 479,126 |
| #17. | learn*:ab,ti | 504,913 |
| #18. | 'recall'/exp | 45,577 |
| #19. | 'attention'/exp | 235,756 |
| #20. | 'recall':ab,ti | 69,678 |
| #21. | attention:ab,ti | 501,091 |
| #22. | 'concentration'/exp | 2,212 |
| #23. | 'concentration':ab,ti | 1,435,211 |
| #24. | 'focus'/exp | 137 |
| #25. | 'focus':ab,ti | 518,168 |
| #26. | 'executive function'/exp | 38,219 |
| #27. | executive AND function:ti,ab | 28,748 |
| #28. | #10 OR #11 OR #12 OR #13 OR #14 OR #15 OR #16 OR  #17 OR #18 OR #19 OR #20 OR #21 OR #22 OR #23 OR  #24 OR #25 OR #26 OR #27 | 4,997,350 |
| #29. | #9 AND #28 | 44,113 |
| #30. | #29 AND 'randomized controlled trial'/de AND ('Article'/it OR 'Article in Press'/it OR  'Conference Abstract'/it OR 'Conference Paper'/it  OR 'Review'/it | 1,603 |

**PubMed:**

Date: Feb 2020, Updated: 25 January 2021

| **No.** | **Query** | **Results** |
| --- | --- | --- |
| #1 | (((((((((anticholinergic*[Title]) OR anticholinergic*[Title/Abstract]) OR cholinergic antagonist[Title]) OR cholinergic antagonist[Title/Abstract]) OR cholinolytic*[Title]) OR cholinolytic*[Title/Abstract]) OR antimuscarinic*[Title]) OR antimuscarinic*[Title/Abstract])) | 16,743 |
| #2 | ((((((executive function[Title]) OR executive function[Title/Abstract])) OR ((((((((((((((((mental state[Title]) OR mental state[Title/Abstract]) OR cognit*[Title]) OR cognit*[Title/Abstract]) OR memory[Title]) OR memory[Title/Abstract]) OR learn*[Title]) OR learn*[Title/Abstract]) OR recall[Title]) OR recall[Title/Abstract]) OR attention[Title]) OR attention[Title/Abstract]) OR concentration[Title]) OR concentration[Title/Abstract]) OR focus[Title]) OR focus[Title/Abstract]))) OR (((((("Cognition"[Mesh]) OR "Memory"[Mesh]) OR "Learning"[Mesh]) OR "Mental Recall"[Mesh]) OR "Attention"[Mesh]) OR "Executive Function"[Mesh])) | 3,153,723 |
| #3 | #1 AND #2 | 3,119 |

**Cochrane** **Library**:

| ID | Search | Hits |
| --- | --- | --- |
| #1 | (cholinergic antagonist):ti,ab,kw | 272 |
| #2 | (anticholinergic*):ti,ab,kw | 3207 |
| #3 | (cholinolytic*):ti,ab,kw | 15 |
| #4 | (antimuscarinic*):ti,ab,kw | 728 |
| #5 | #1 OR #2 OR #3 OR #4 | 4056 |
| #6 | (executive function):ti,ab,kw | 4768 |
| #7 | (mental state):ti,ab,kw | 8597 |
| #8 | (cognit*):ti,ab,kw | 72230 |
| #9 | (memory):ti,ab,kw | 22249 |
| #10 | (learn*):ti,ab,kw | 29907 |
| #11 | (recall):ti,ab,kw | 9250 |
| #12 | (attention):ti,ab,kw | 29001 |
| #13 | (concentration):ti,ab,kw | 94765 |
| #14 | (focus):ti,ab,kw | 22682 |
| #15 | MeSH descriptor: [Cognition] explode all trees | 9738 |
| #16 | MeSH descriptor: [Memory] explode all trees | 7297 |
| #17 | MeSH descriptor: [Mental Recall] explode all trees | 2357 |
| #18 | MeSH descriptor: [Attention] explode all trees | 5151 |
| #19 | MeSH descriptor: [Executive Function] explode all trees | 917 |
| #20 | #6 OR #7 OR #8 OR #9 OR #10 OR #11 OR #12 OR #13 OR #14 OR #15 OR #16 OR #17 OR #18 OR #19 | 234940 |
| #21 | #5 AND #20 | 869 |

**Scopus**:

| Query | Results |
| --- | --- |
| TITLE-ABS-KEY(anticholinergic* OR cholinergic antagonist OR cholinolytic* OR antimuscarinic*) AND TITLE-ABS-KEY((executive function) OR mental state OR cognit* OR memory OR learn*OR recall OR attention OR concentration OR focus) AND ( LIMIT-TO ( DOCTYPE,"ar" ) OR LIMIT-TO ( DOCTYPE,"re" ) OR LIMIT-TO ( DOCTYPE,"cp" ) ) AND ( LIMIT-TO ( SRCTYPE,"j" ) ) | 217 |

**Web of Science**

| **Set** | **Results** | **Save History / Create AlertOpen Saved History** |
| --- | --- | --- |
| # 3 | [**317**](http://apps.lib.wosg.ir/summary.do;jsessionid=D457B3483A55FA92F76EF1EAE7C97F6A?product=WOS&doc=1&qid=6&SID=6Cw6J91ajKy2llJCVIM&search_mode=GeneralSearch&update_back2search_link_param=yes) | **TITLE:** ("executive function" OR mental state OR cognit* OR memory OR learn*OR recall OR attention OR concentration OR focus) *AND* **TITLE:**(anticholinergic* OR cholinergic antagonist OR cholinolytic* OR antimuscarinic*)  *Indexes=SCI-EXPANDED, SSCI, A&HCI, CPCI-S, CPCI-SSH, BKCI-S, BKCI-SSH, ESCI, CCR-EXPANDED, IC Timespan=All years* |
| # 2 | [**725**](http://apps.lib.wosg.ir/summary.do;jsessionid=D457B3483A55FA92F76EF1EAE7C97F6A?product=WOS&doc=1&qid=4&SID=6Cw6J91ajKy2llJCVIM&search_mode=GeneralSearch&update_back2search_link_param=yes) | **TOPIC:** ("executive function" OR mental state OR cognit* OR memory OR learn*OR recall OR attention OR concentration OR focus) *AND* **TITLE:**(anticholinergic* OR cholinergic antagonist OR cholinolytic* OR antimuscarinic*)  **Refined by:** **DOCUMENT TYPES:** ( ARTICLE OR MEETING ABSTRACT OR REVIEW OR PROCEEDINGS PAPER )  *Indexes=SCI-EXPANDED, SSCI, A&HCI, CPCI-S, CPCI-SSH, BKCI-S, BKCI-SSH, ESCI, CCR-EXPANDED, IC Timespan=All years* |
| # 1 | [**767**](http://apps.lib.wosg.ir/summary.do;jsessionid=D457B3483A55FA92F76EF1EAE7C97F6A?product=WOS&doc=1&qid=3&SID=6Cw6J91ajKy2llJCVIM&search_mode=GeneralSearch&update_back2search_link_param=yes) | **TOPIC:** ("executive function" OR mental state OR cognit* OR memory OR learn*OR recall OR attention OR concentration OR focus) *AND* **TITLE:**(anticholinergic* OR cholinergic antagonist OR cholinolytic* OR antimuscarinic*)  *Indexes=SCI-EXPANDED, SSCI, A&HCI, CPCI-S, CPCI-SSH, BKCI-S, BKCI-SSH, ESCI, CCR-EXPANDED, IC Timespan=All years* |
|  |  |  |
