# Supplementary material for: Cognitive effects of individual anticholinergic drugs: a systematic review and meta-analysis
Source: Dement Neuropsychol. 2023 May 29;17:e20220053. doi: 10.1590/1980-5764-DN-2022-0053 (PMC10229087; doi:10.1590/1980-5764-DN-2022-0053)
Supplement: Supplementary file 2 [file 1980-5764-DN-17-e20220053-Suppl02.docx]

**Supplementary Material 2**

The characteristics and summary of findings of included articles.

| **Study** | **Study Design** | **Condition** | **Sample size** | **Mean age ± SD/SEM (Range)** | **Number of males** | **Cognitive test** | **Drug-test interval** | **Route of administration** | **Anticholinergic drug** | **Drug Consumption** | **Outcome** |
| --- | --- | --- | --- | --- | --- | --- | --- | --- | --- | --- | --- |
| Atri 2004 [1] | CT | Volunteer | 28 | 21  (18-29) | 9 | Standard cued recall, Free Recall | - | Intramuscular | Scopolamine HBr  Glycopyrrolate | 8 µg/kg  and 4 µg/kg | *Compared with glycopyrrolate and subjects who were not injected, scopolamine showed overall impairment in new word paired-associate learning, but no impairment in cued recall of previously learned associates; and greater impairment in learning overlapping compared with nonoverlapping paired associates.* |
| Baakman 2017 [2] | Double-blind crossover CT | Volunteer | 12 | 25.9  (19-36) | 12 | Halstead-Reitan Test Battery, Adaptive tracking test, Simple reaction time task, The Visual Verbal Learning Test | 200-300 min | Oral  Intravenous | Mecamylamine  Scopolamine HBr | 10, 20 mg  and 0.5 mg | *Mecamylamine had a dose dependent effect decreasing the adaptive tracking performance and visual analog scale alertness, and increasing the visual verbal learning task performance time and errors. Scopolamine significantly affected almost all tests. These effects last up to 10 hours after drug administration.* |
| Beatty 1986 [3] | Double-blind crossover CT | AD, HD, Volunteer | 6 | 26  (20-32) | 6 | Free recall, Verbal fluency, Delayed recall and recognition, Symbol-digit paired-associate learning, Brown-Peterson | 0-105 min | Intramuscular | Scopolamine  Glycopyrrolate | 0.5 and  0.5 mg | *Scopolamine impaired acquisition and delayed recall of wordlist and disrupted retention on the Brown-Peterson distract or task, whereas glycopyrrolate was without effect. Scopolamine did not increase the number of false positive errors on delayed recognition of the word list and also failed to increase the number of prior item intrusions on the Brown-Peterson task. scopolamine did not impair learning of a symbol digit paired-associate task, and did not reduce the number of words retrieved or increase the number of words repeated on a standardized verbal fluency test.* |
| Bishop 1996 [4] | Double-blind RCT | Volunteer | 60 | 25  (18-41) | 30 | Digit Span, Mental rotation, Logical reasoning, Sentence verification, word-stem completion task, immediate and delayed recall, Digital cancellation | 1-2 hours | Injection | Scopolamine | 0.3, 0.6 mg | *The effects of Scopolamine were qualitatively indistinguishable on tasks tapping working, episodic, semantic, and procedural memory. Scopolamine did not affect word-stem completion.* |
| Borghans 2017 [5] | Double-blind crossover CT | Volunteer | 17 | 22.4±3.0 | 7 | Verbal learning task, immediate and delayed recall, Verbal recognition task | 1 hour | Oral | Biperiden | 2 mg | *Results revealed decreased scores on the delayed recall after biperiden and acute tryptophan depletion separately but no significant interaction between the two.* |
| Borghans 2020 [6] | double-blind crossover CT | Volunteer | 21 | 22.6±3.7 | 5 | Rey auditory verbal learning test, N-back task, choice reaction task, incongruent choice task, sustain attention task | 90-110 min | Oral | Biperiden | 2, 4 mg | *Biperiden decreased the number of words recalled in immediate and delayed recall of the verbal learning task 90 minutes after drug intake. A dose-dependent impairment was found for the delayed recall, whereas the immediate recall was equally impaired by the 2 doses. Biperiden did not affect the performance on the verbal learning task 4 hours after administration. Performance in the n-back task and the sustained attention to response task were not affected by biperiden at any time point.* |
| Brandeis 1992 [7] | double-blind crossover CT | Volunteer | 12 | -  (21-40) | 7 | Selective reminding test, attention (ERP task) | - | - | Scopolamine HBr | 1.2 mg | *Scopolamine slowed latency in all tasks, and reaction time in some tasks. Visual processing was affected.* |
| Broks 1988 [8] | double-blind crossover CT | Volunteer | 20 | -  (18-48) | 10 | Selective and sustained attention, digit and spatial span, verbal and spatial recall, Visual contrast sensitivity, choice reaction time | - | Oral | Scopolamine,  methyl scopolamine | 0.3, 0.6, 1.2 mg | *While some functions were unaffected by the drug (e.g., alerting) and others were impaired at the highest dose (e.g., verbal learning) still others were affected in a linear dose-dependent manner (sustained attention; visual contrast sensitivity).* *In no case did methyl-scopolamine produce effects that differed significantly from placebo.* |
| Brown 2015 and 2016 [9, 10] | crossover RCT | Volunteer | 18 | 21  (18-26) | 3 | attentional blink task, Simple reaction time task, Classic oddball, Novelty oddball, multiple target detection | 90-120 min | Oral | Scopolamine | 1.2 mg | *Scopolamine may impair temporal attention through a decrease in tonic alertness.* |
| Bukala 2019 [11] | Double blind RCT | Volunteer | 33 | - | - | Emotional recall, masked and unmasked attentional vigilance, | - | Transdermal | Scopolamine | 1.5 mg for 17 h | *There was no clear effect of scopolamine patch on emotional cognition, verbal or working memory, suggesting that the effective dose of scopolamine available through the patch is too low to represent a viable antidepressant mechanism.* |
| Caine 1981 (Experiment I) | Crossover RCT | Volunteer | 9 | 20.8  (19-26) | 9 | Brown-Peterson Task, immediate recall, delayed recall, cued recall | 60 min | Intramuscular | Scopolamine HBr,  Methyl Scopolamine | 0.8, 0.5 mg | *The drug does not diminish attention, as assessed with an auditory vigilance task, or initial signal detection. More complex auditory decoding is affected, however. Scopolamine impairs aspects of initial memory acquisition (e. g., encoding and consolidation) and spontaneous memory retrieval. Retention is unaffected. There were no significant differences between methscopolamine and placebo in any of the tests.* |
| Caine 1981 (Experiment II) | Crossover RCT | Volunteer | 7 | 22  (20-23) | 5 | Words recall, pattern recall | 90 min | Intramuscular | Scopolamine HBr | 0.8-0,6 mg |  |
| Caine 1981 (Experiment III) [12] | Crossover RCT | Volunteer | 7 | 22  (20-23) | 5 | Vigilance task | 120 min | Intramuscular | Scopolamine HBr | 0.8-0,6 mg |  |
| Callaway 1958 [13] | Double blind RCT | Volunteer | 21 | - | - | The Stroop test, Gottschaldt Test | 15 min | Intramuscular | Atropine sulfate | 2 mg | *Atropine was found to improve performance on a task demanding broad attention and to impair performance on tasks demanding a narrower focus of attention.* |
| Callaway 1985 [14] | Double blind RCT | Volunteer | 12 | 25  (19-33) | 0 | Stimulus evaluation response selection task, Sternberg fixed set memory scanning task, Automated span of apprehension test, Rapid execution of long and short sequences | 45 min | Oral | Scopolamine HBr | 0.6, 1.2 mg | *Scopolamine slowed RT and P300 as had age, but scopolamine slowed responses to simple stimuli more than responses to complex stimuli. Scopolamine effects on other tasks in the battery were small but consistent with an action of scopolamine on an early stimulus preprocessing stage that is independent of a stimulus evaluation stage that is also affected by age.* |
| Chhatwal 2018 [15] | double-blind crossover RCT | Volunteer | 243 | Scopolamine group: 73.8±6.7  Control group: 73.9±6.1 | 101 | MMSE, Buschke Selective reminding task | 5-30 min | Intravenous | Scopolamine | 0.2 mg | *Low-dose scopolamine decreased episodic memory performance.* |
| Craig 2010 [16] | Crossover CT | Benign leiomyoma uteri | 30 | 40.4±4.65 | 0 | CANTAB Delayed Match to Sample task | 1 hour | Subcutaneous | Scopolamine | 0.6 mg | *Following pharmacological-induced menopause, cholinergic depletion produces a more significant behavioral deficit in overall memory performance, as manifest by increased response time.* |
| Crow 1971 and 1973 [17, 18] | Double-blind crossover CT | Volunteer | 12 | 19-23 | 10 | Immediate recall, delayed recall, number-color association test, vigilance task, scanning task | - | Intravenous | Scopolamine  Atropine | 0.4 and  0.6 mg | *Subjects receiving hyoscine showed a significant reduction in performance on both the delayed recall and the number-color association tests, but no reduction in the vigilance task, and a much smaller reduction in the immediate recall test. There was no impairment after treatment with atropine.* |
| Crow 1975 [19] | Double blind CT | Volunteer | 8 | 19-23 | 8 | Immediate recall, delayed recall, scanning task | - | Intravenous | Scopolamine | 0.4 mg | *Scopolamine administration leaves scanning task performance and immediate recall unaffected but significantly impairs delayed recall.* |
| Curran 1991 [20] | Double blind RCT | Volunteer | 36 | 27  (21-49) | 12 | Digit span, Mental rotation, Baddeley reasoning test, Paired-associate interference task, Prose recall, Word fluency, symbol copying test, digit symbol substitution test | 1 hour | Intramuscular | Scopolamine | 0.3, 0.6 mg | *The only significant difference found on digit span task was between the two doses of scopolamine whereby 0.6mg slightly reduced digit span at the1-h testing time compared with 0.3 mg. Accuracy on mental rotation task was not affected with treatment. Subjects given scopolamine showed little change in response times in Baddeley reasoning test. Immediate recall data showed that all active treatments impaired performance compared with placebo and the effect of scopolamine was dose related and greatest at 1 h in prose recall.* |
| Curran 1998 [21] | double-blind CT | Volunteer | 50 | 28.2  (20-46) | 25 | Immediate recall, intertap interval | 1 hour | Subcutaneous | Scopolamine | 0.6 mg | *Scopolamine produced marked impairments of episodic memory. Memory impairments by Scopolamine were highly significant on retention in the continuous recognition task and further, no drug effects were found on response bias. Subsequent free recall was similarly very impaired by Scopolamine.* |
| Danion 1990 [22] | Double blind RCT | Volunteer | 48 | 23  (20-27) | - | Digit symbol substitution task, Word recall | 1 hour | Intramuscular | Scopolamine HBr | 6 µg/kg | *No significant changes in memory performances were observed in the scopolamine group; however, a significant correlation between explicit and implicit memory performances was observed in this group.* |
| Diefenbach 2003 [23] | double-blind, crossover RCT | Volunteer | 24 | 28.5±5 | 18 | Zahlen-Verbindungs Test, d2 attention test | 1 hour | Oral | trospium chloride  Tolterodine  Oxybutynin | 45 mg  4 mg  15mg | *The number combination test (ZVT), the primary parameter of cognitive function, and the d2 test did not reveal any differences in reaction time.* |
| Diefenbach 2005 [24] | double-blind RCT | Volunteer | 24 | 60±3 | 12 | Zahlen-Verbindungs Test, d2 attention test | 1 hour | Oral | trospium chloride  Tolterodine  Oxybutynin | 45 mg  4 mg  15mg | *There was no effect of the tested anticholinergics on cognitive variables.* |
| Drachman 1974 [25] | CT | Volunteer | 43 | 22.8  (19-25) |  | Digit span, free recall, retrieval by category test, performance and verbal and full-scale IQ, Organicity Index | 1 hour | Subcutaneous | Scopolamine  Methscopolamine | 1 mg  1 mg | *Subjects receiving scopolamine*  *showed impairment of memory storage and possibly retrieval despite normal immediate memory span; non memory cognitive functions were also impaired. Methscopolamine did not produce any significant changes in memory or other cognitive functions.* |
| Dubeau 2014 [26] | Double-blind RCT | subjects with urgency urinary incontinence (MMSE>20) | 562 | Fesoterodine group: 74.8 (65-91)  Control group: 75.3  (65-90) | 101 | MMSE | - | Oral | Fesoterodine | 4, 8 mg once daily for 12 weeks | *There was no deterioration in mean MMSE scores from baseline to week 12 in either group. Subjective memory impairment was reported in 2 subjects treated with Fesoterodine with onset after increase to the 8 mg dose.* |
| Duka 1992 [27] | Double blind RCT | Volunteer | 40 | 25  (18-42) | 40 | Verbal memory test | 45-60 min | Subcutaneous | Scopolamine | 0.5 mg | *Scopolamine did not impair delayed recall of visual or verbal material.* |
| Duka 1995 and 1996 [28, 29] | double-blind RCT | Volunteer | 36 | -  (21-38) | - | Logical reasoning, Continuous attention task, Visual vigilance task, Rapid information processing, Visual memory test | 1.5-2 hours | Subcutaneous | Scopolamine | 0.5 mg | *Scopolamine impaired performance in attentional and vigilance tasks as well as in the rapid information processing task significantly. Scopolamine did not have any effect on in the logical reasoning task. Scopolamine impaired performance in the immediate recall but left delayed recall unaffected. These data suggest that scopolamine at this dose impaired mostly attention and early stages of information processes. Scopolamine significantly impaired performance in attentional and vigilance tasks.* |
| Dundee 1972 [30] | Double blind RCT | scheduled for minor gynecological operations | 160 | reproductive years | 0 | Recognition | 60 min | Intravenous | Scopolamine HBr | 0.6 mg | *Hyoscine caused amnesia in 35 and 50% of patients with peak effect not occurring until 50-80 min after injection and action persisting for at least 120 minutes.* |
| Dunne 1985 [31] | Double-blind crossover CT | Volunteer | 14 | - | 0 | Immediate recall task, delayed recall task, recognition task | 1-2 hours | Oral | Scopolamine HBr | 0.6 mg | *While there was no drug effect upon total recall, there was a significant drug by attention interaction, in that recall for attended words was impaired while unattended recall was facilitated. There were no drug effects upon recognition performance.* |
| Dunne 1986 [32] | Double blind crossover CT | Volunteer | 20 | 27  (19-40) | 0 | Target detection | 50 min | Oral | Scopolamine HBr | 0.3 mg x 3 | *Results showed a significant interaction between drug condition and target location probability, that offer support for the notion that scopolamine reduces the efficiency of information encoding because it impairs the optimal utilization of attentional resources.* |
| Dunne 1990 [33] | Double blind crossover CT | Volunteer | 14 | 26  (18-37) | 7 | Continuous retrieval | 50 min | Oral | Scopolamine HBr | 0.3 mg x 3 | *Scopolamine increased the frequency of item repetition, but had no effect on the number of items subjects could retrieve, or the number of items retrieved per retrieval.* |
| Dunne 1993 [34] | Double-blind crossover CT | Volunteer | 18 | 36.4±10.1 | 18 | NART IQ, Total short-term recall, Delayed recall, Digit span, Benton VRT, Warrington faces, Letter and Number cancellation, Symbol digit substitution, Block design, Verbal fluency | 45 min | Subcutaneous | Scopolamine | 0.4 mg | *Performance was poorer following scopolamine on four tests of visuomotor speed and problem solving. These were letter cancellation, number cancellation, symbol- digit substitution and block design. In addition, there were non-significant trends for poorer performance under the drug in delayed recall and the Trails B test. In contrast to this general pattern of impairment, the drug produced a significant improvement in the verbal fluency test.* |
| Ebert 1998 [35] | double-blind  crossover RCT | Volunteers | 10 | 24  (22-25) | 10 | Alertness, number matching task, Immediate recall | 1 hour | Subcutaneous | Scopolamine HBr | 0.4,  0.6,  0.8 mg | *Scopolamine produced dose- and time-dependent impairments of attention and memory.* |
| Edginton 2003 [36] | double-blind RCT | Volunteers | 30 | 23  (18-42) | 11 | different word sets recalled | 1 hour | Subcutaneous | scopolamine | 0.3,  0.5 mg | *Consistent with an effect on encoding, scopolamine reduced recall for all. Scopolamine did not differentially affect practiced or unpracticed exemplars, relative to the control words.* |
| Ellis 2006 [37] | double-blind crossover RCT | Volunteers | 12 | 22.4±2.4 | 10 | Cognitive Drug Research computerized assessment | 2-3 hours | Oral (mecamylamine), intramuscular (scopolamine) | Mecamylamine,  scopolamine | 15 mg and  0.4 mg | *Muscarinic receptor antagonism with scopolamine resulted in deficits in working memory, declarative memory, sustained visual attention and psychomotor speed. Nicotinic antagonism with mecamylamine had no effect on any of the cognitive processes examined. Simultaneous antagonism of both muscarinic and nicotinic receptors with mecamylamine and scopolamine impaired all cognitive processes impaired by scopolamine and produced greater deficits than either muscarinic or nicotinic blockade alone, particularly on working memory, visual attention and psychomotor speed.* |
| Erskine 2004 [38] | double-blind crossover RCT | Volunteers | 12 | 23.5±3.232 | 10 | Inspection time | 2-3 hours | Oral (mecamylamine), intramuscular (scopolamine) | Mecamylamine,  scopolamine | 15 mg and  0.4 mg | *Selective blockade of nicotinic receptors with mecamylamine did not significantly impair Inspection time, whereas selective blockade of muscarinic receptors with scopolamine produced a significant but small impairment in Inspection time. Combined blockade of both receptor types with scopolamine and mecamylamine produced a large impairment in Inspection time performance.* |
| Flicker 1990 [39] | Crossover CT | Volunteers | 30 | -  (18-30) | - | Digit span, Delayed recall, Object naming, Recognition, Object sorting, Digit symbol, Driving test travel | 30 min | Subcutaneous | Scopolamine HBr | 0.43 mg/ 70 kg | *Scopolamine produced deficits on tests of verbal recall, visuospatial recall, visual recognition memory, visuospatial praxis, Visuoperceptual function, and psychomotor speed. Immediate memory, language function, object sorting, and frequency of intrusion errors were unaffected. The low dose of scopolamine produced*  *some peripheral anticholinergic signs but did not affect the cognitive measures.* |
| Flicker 1992 [40] | Crossover CT | Volunteers | 20 | Young: 23.1±4.7  Elderly: 70.0±6.3 | 9 | Digit span, Object naming, recognition | 30 min | Subcutaneous | Scopolamine HBr | 0.43 mg/ 70 kg | *scopolamine significantly impaired performance on tests of recent memory and visuospatial praxis. The same effects were observed in the elderly subjects, but the magnitude of the effects was much larger. The scopolamine injections produced significant psychomotor slowing in the elderly, whereas higher doses of the drug are required to produce this effect in young subjects. In both young and old subject’s scopolamine failed to affect immediate memory, language function, object sorting, and the frequency of intrusion errors. Remote memory, tested in the elderly only, was also unaffected.* |
| Fredrickson 2008 [41] | double-blind Crossover RCT | Volunteers | 24 | 32.6±4.5 | 24 | CogState (Psychomotor function, Attention, Visual learning, Executive function, Delayed memory recall) | 2 hours | Subcutaneous | scopolamine | 0.2, 0.4,  0.6 mg | *Scopolamine significantly impaired performance on all tasks in a dose and time related manner. These results demonstrate the functionality of change scores to draw comparisons between different times and doses.* |
| Frith 1984 [42] | Double blind CT | Volunteers | 9 | -  (19-51) | 6 | Immediate Serial Recall | 5-10 min | Intravenous | Scopolamine | 5.7 µg/kg | *Hyoscine produced deficits on concrete and abstract words whether scored for ordered recall or item recall. In terms of ordered recall, phonemic similarity produced impaired performance but semantic similarity did not. In terms of item recall, hyoscine produced impaired performance on unrelated words, but the impairment was reduced under conditions of either phonemic or semantic similarity.* |
| Frith 1989 [43] | RCT | Volunteers | 28 | -  (18-50) | - | target tracking task | 10-30 Min | Intravenous | Scopolamine | 0.4 mg | *General impairment was seen 20 min later in significantly decreased tracking performance. Subjects treated with scopolamine, showed only slow temporary improvement and little permanent improvement in their performance at this task.* |
| Geller 2017 [44] | double-blind RCT | OAB | 45 | Trospium group: 66.7±10.1  Control group: 68.9±10.9 | 0 | MMSE, Trails A, Digit Span, HVLT-R, Mini Mental Status X | At the peak plasma concentration | Oral | trospium chloride XR | 60 mg daily for 4 weeks | *For the primary outcome, there was no difference in HVLT-R total score between trospium and placebo groups at week 4. There were also no differences based on the other cognitive tests. There was a correlation between age and the following week-4 tests: HVLT-R total score, HVLT-R total recall subscale, Trails A, and Trails B.* |
| Ghoneim 1975 [45] | double blind crossover CT | Volunteers | 36 | 21  (19-27) | 18 | immediate recall, Delayed recall, Recognition | 90 min | Intramuscular | Scopolamine | 8 μg/kg | *Scopolamine impaired memory functions. The deficit appeared to be in the storage process leaving retrieval processes unaffected.* *Scopolamine in addition interfered with organizational processes.* |
| Ghoneim 1977 [46] | double blind crossover CT | Volunteer | 70 | 23  (19-32) | 35 | immediate recall, Delayed recall, Recognition | 35-170 min | Intramuscular | Scopolamine | 8 μg/kg | *Scopolamine did not affect recall of information which had been learned prior to drug injection. However, impaired the learning or acquisition of new information.* |
| Giramonti 2008 [47] | double blind crossover RCT | urgency or urge incontinence | 14 | 7.7  (5-11) | 9 | Developmental Neuropsychological Assessment (NEPSY) | - | Oral | Tolterodine  Oxybutynin | 0.1 0.3 mg/kg for 2 weeks | *Attention and memory scores increased over time in all children, however, the analyses showed no significant negative effects of anticholinergic medications on Attention or Memory.* |
| Golding 1989 [48] | Crossover CT | Volunteer | 12 | (18-30) | 12 | Four Choice Reaction Time (4CRT), Mean Target Tracking Error, Letter cancellation, logical reasoning | 1-3 hours | Oral | Scopolamine HBr | 1.2 mg | *Hyoscine impaired performance 1-3 hours post drug.* |
| Golding 2018 [49] | double-blind Crossover RCT | Volunteer | 16 | 26.47±7.94 | 16 | psychomotor cognitive function | 90 min | Oral | Scopolamine HBr, Darifenacin | 0.6 mg, 10, 20 mg | *Darifenacin produced either no effect or an enhanced effect on cognitive function in contrast to Hyoscine hydrobromide where there was significant impairment of psychomotor performance.* |
| Gordon 2001 [50] | double-blind crossover RCT | Volunteer | 60 | -  (18-20) | - | Digit span, Trail making, Decision Time, Motor Time, word recall, digit symbol substitution, signal detection time | 12 hours | Transdermal | Scopolamine | 1.5 mg | *Transdermal scopolamine did not affect performance abilities.* |
| Grasby 1995 [51] | Single blind CT | Volunteer | 12 | -  (21-36) | 12 | Word recall | - | Subcutaneous | Scopolamine | 0.4 mg | *Scopolamine attenuated memory task-induced increases of regional cerebral blood flow in the left and right prefrontal cortex and the right anterior cingulate region.* *Scopolamine had no effect on the number of words correctly recalled in the five-word list task.* *Scopolamine reduced the numbers of words correctly recalled in the 15-word list task compared to placebo* |
| Green 2005 [52] | double-blind crossover RCT | Volunteer | 12 | 23.3±2.8 | 10 | Object 1&2-back  Accuracy, Object 1&2-back Reaction time, spatial 1&2-back Accuracy, Spatial 1&2-back Reaction Time | 2-3 hours | Oral (mecamylamine), intramuscular (scopolamine) | Mecamylamine, Scopolamine | 15 mg  0.4 mg | *Both muscarinic and nicotinic receptors may functionally interact to synergistically modulate n-back working memory.* |
| Grober 1989 [53] | Double blind RCT | Volunteer | 12 | -  (22-35) | 12 | Free recall, Total recall | - | - | Scopolamine | 0.3, 0.5, 1 mg | *Cholinergic blockade may impair memory indirectly through effects on other cognitive processes.* |
| Harel 2013 [54] | double-blind crossover RCT | Volunteer | 34 | 31.8±6.9 | 34 | Continuous PAL | 2 hours | subcutaneous | Scopolamine | 0.6 mg | *Acute scopolamine challenge can produce large and robust deficits in visual–spatial PAL, which reflect impairments in both memory and executive processes.* |
| Hardy 1962 [55] | Comparative trial | Surgical candidates | 200 | -  (15-65) | - | Picture recall |  | Subcutaneously | Scopolamine Atropine | 0.4  0.6 | *seven patients out of the hundred who had hyoscine had no memory of the picture shown to them. However, all the patients who had atropine remembered the picture.* |
| Higgins 1989  [56] | Crossover CT | Volunteer | 7 | 26±3.7 | 7 | multiple schedules of repeated acquisition | 0.5, 1.5, 3.0, 5.0, 7.0, 9.0, and 24.0 hours | Intramuscular | Atropine | 1.5, 3.0, 6.0 mg/70 kg | *Overall percentage of errors increased and overall response rates decreased in the acquisition and performance components as an orderly function of drug dose. However, these effects were selective in that behavior in the acquisition component generally was affected at lower doses than in the performance component. None of the drug doses produced reliable effects the day after drug administration (24- hours post drug) in either schedule component.* |
| Hongyu 2019  [57] | RCT | Patients with lung cancer undergoing thoracoscopic surgery | 90 | 65-80 | 47 | MMSE, Postoperative cognitive dysfunction rate | 1, 4 and 7 day after surgery | Intramuscular | Atropine | 0.01mg/kg | *There was no significant difference in preoperative and day 1 post-surgery MMSE scores.* |
| Howland 2008  [58] | double-blind crossover RCT | Volunteer | 32 | 28.1±5.4 | 31 | Psychomotor Vigilance Task  Median Reaction Time | - | transdermal | scopolamine | 1.5 mg | *Transdermal scopolamine patch does not impair simulated ship handling.* *There were no significant differences on occupational outcomes by medication condition, but sustained reaction time was significantly increased under transdermal scopolamine, relative to placebo.* |
| Jacob Huff 1988  [59] | CT | Volunteer | 6 | 63.8  (54-72) | 4 | Word list learning, Continuous performance, Category fluency, Naming to definition, Form discrimination | 30 min | intramuscular | scopolamine | 0.1, 0.2 mg | *Subjects showed dose-related impairments in verbal learning and scopolamine did not alter retrieval from long-term lexical-semantic memory or performance on a test of visual discrimination, suggesting that cholinergic neurotransmission is not critical for these cognitive functions.* |
| Jones 1979  [60] | double-blind RCT | Volunteer | 102 | - | - | short-term retention of digit strings and the free recall of items from categorizable lists | 90 min | Oral | Scopolamine HBr | 0.3 mg | *3 The short-term, retention of digits was impaired to an equivalent degree and locus for hyoscine. This effect was ascribed to the action on primary memory. The drug produced no significant effects on the recall of categorizable lists either in terms of the number of words recalled or the cohesiveness of categorical recall.* |
| Kamboj 2006a  [61] | double-blind RCT | Volunteer | 48 | 23±4 | 24 | Recognition memory (emotional & neutral) | 1 hour | Subcutaneous | scopolamine | 0.6 mg | *the emotional memory advantage was retained for recall and recognition memory under placebo conditions. However, lorazepam and scopolamine produced anterograde recognition memory impairments on both the neutral and emotional components of the narrative, although floor effects were obtained for recall memory.* |
| Kamboj 2006b  [62] | double-blind RCT | Volunteer | 48 | 23 | 24 | Immediate explicit memory, Delayed explicit memory | 1 hour | Subcutaneous | scopolamine | 0.6 mg | *Scopolamine-induced cholinergic hypo- function selectively impaired the recognition accuracy of disgust and anger facial expressions.* |
| Katz 1998  [63] | double-blind crossover RCT | Volunteer | 12 | 69.17±3.83 | 7 | Buschke Selective Reminding Test, Digit span, Verbal fluency, Digit symbol substitution, Trail making, Contingent continuous performance, Recognition and recall pattern | 90 min | Oral | oxybutynin hydrochloride | 5, 10 mg | *Oxybutynin caused significant cognitive decrements on seven of 15 cognitive measures. The most sensitive measures for detecting the effects of oxybutynin hydrochloride were the Buschke Selective Reminding Test and Reaction Time.* |
| Kay 2005  [64] | double-blind crossover RCT | Volunteer | 27 | 28  (19-44) | 27 | Simple reaction time, Speed of spatial and numeric working memory, Sensitivity and speed of picture recognition, HVLT-R | 2 hours | Oral | Darifenacin  Dicyclomine | 7.5, 15 mg  20 mg | *Compared with placebo, neither dose of darifenacin affected cognitive function, whereas dicyclomine impaired performance on five of the 12 variables 2 h after dosing; simple reaction time, speed of numeric and spatial working memory, and speed and sensitivity of picture recognition.* |
| Kay 2006  [65] | double-blind RCT | Volunteer | 150 | >60 |  | delayed recall on Name-Face association, delayed recall on First-Last name association | - | Oral | Darifenacin,  Oxybutynin | 7.5 mg  15 mg | *While darifenacin had no significant effects on memory versus placebo, oxybutynin ER caused significant memory deterioration. The results also demonstrate that subjects may not recognize/report memory deterioration.* |
| Kay 2012a  [66] | double-blind crossover RCT | Volunteer | 20 | 72.2±5.2 | 12 | Detection task, Identification Task, One Card Learning Task, Continuous PAL, Groton Maze Learning Task, Rey Auditory Verbal Learning Test, HVLT-R | - | - | Fesoterodine | 4, 8 mg | *In healthy older adults, Fesoterodine 4 and 8 mg once daily had no statistically significant effects versus placebo on any cognitive function assessed, including memory.* |
| Kay 2012b  [67] | Double-blind RCT | Volunteer | 152 | 68.2±5.76 | 53 | Delayed Recall and Learning Trial, Facial Recognition, HVLT-R, free recall, Memory Assessment Clinics Self-Report Questionnaire | 1 hour | Transdermal (topical gel) and oral (IR capsule) | Oxybutynin chloride | 1 g  5 mg | *Oxybutynin topical gel applied for 1 week had no clinically meaningful effect on recent memory or other cognitive functions in healthy, older adults. Oral IR Oxybutynin failed to show a decline on the primary end point (NFAT Delayed Recall scores); however, significant declines were found on other measures of recent memory.* |
| Klinkenberg 2012  [68] | Double-blind crossover CT | Volunteer | 17 | 22.4±3 | 7 | learned irrelevance index | 1 hour | Oral | Biperiden | 2 mg | *Biperiden had no effect on the behavioral learned irrelevance index measures, although prolonged reaction times were evident.* |
| Koller 2003  [69] | double-blind crossover RCT | Volunteer | 12 | 23.8±2.2 | 4 | Immediate and delayed free recall, visual, word and picture recognition, matching to sample, continuous performance test, span of apprehension | - | Subcutaneous | Scopolamine HBr | 0.3, 0.6 mg | *Significant impairment by both doses of scopolamine was seen in immediate and delayed free recall, continuous visual recognition, running word recognition and running picture recognition. scopolamine has a greater effect on memory than on attention.* |
| Kosilov 2018  [70] | Cohort RCT | urge or mixed urinary incontinence | 312 | 69.4  (60-83) | 0 | MMSE, Controlled Oral Word Association Test, Wechsler Adult Intelligence Scale-Revised Digit span, The Color Trails Test, Wechsler Memory Scale, California Verbal Learning Test, | - | - | Solifenacin + trospium | 10+30 mg, 20+60 mg | *The cognitive function parameters did not differ at the start and end of the study across the groups. Additionally, the cognitive function parameters did not differ significantly within each group between the start and end of the study.* |
| Lavoie 2019  [71] | double-blind RCT | COPD | 304 | 65±6.6 | 200 | Montreal Cognitive Assessment (MoCA) Test | - | Respimat inhaler | Tiotropium | 5 µg (once daily for 12 weeks) | *There were no significant between-group differences in any outcome with the exception of cognitive function. Greater improvements in MoCA were seen in patients who exhibited greater increases in physical activity and exercise capacity.* |
| Liem-moolenaar 2011  [72] | double-blind crossover CT | Volunteer | 90 | 18-55 | 90 | Stroop test, Visual Verbal Learning Test (immediate recall, delayed recall, delayed word recognition) | 2-3 hours | Intravenous | scopolamine | 0.5 mg | *Memory performance deteriorated after scopolamine for the third immediate recall trial and delayed recall. The average reaction time of the delayed word recognition test longer after scopolamine. The number of correct responses for this same test decreased.* |
| Lines 1991  [73] | CT | Volunteers | 18 | 31  (18-46) | 9 | Verbal fluency, Categorization latency | 45 min | Oral | scopolamine | 0.6, 1.2 mg | *No evidence for a scopolamine deficit in semantic retrieval was found; in fact, scopolamine improved letter fluency. However, scopolamine did produce the expected decrease in visual contrast sensitivity.* *The doses of scopolamine used here have also been shown to impair learning and attention.* |
| Lipton 2005  [74] | double-blind crossover RCT | Volunteers (with no/mild cognitive impairment) | 129 | -  (≥65) | 54 | computer assisted cognitive function tests | - | Oral | Darifenacin | 3.75, 7.5, 15 CR (once per day), 5 IR (3 times daily) | *In speed of choice reaction time and word recognition sensitivity, there were no statistically significant. There were no significant differences in secondary variables except memory scanning speed, which increased in all groups relative to baseline, but improvement was greater with placebo than with 3.75 mg darifenacin.* |
| Little 1995  [75] | crossover double-blind RCT | Volunteers | 10 | 65.2±7.8 | 4 | Episodic memory, Semantic memory, Lexical search &retrieval  Fluency, Processing speed | 70 min | Intravenous | scopolamine | 0.4mg | *Scopolamine had major cognitive effects across a host of individual tests such as list learning and processing speed. For the parallel visual search comparison, subjects performed significantly more slowly on scopolamine compared to placebo. Measures of letter fluency revealed a trend towards impairment with scopolamine and it diminished list learning significantly.* |
| Little 1998  [76] | double-blind RCT | Volunteers | 8 | 61.9±8.3 | 3 | Episodic memory, Semantic memory, Lexical search &retrieval  Fluency, Processing speed, Explicit Memory | 75 min | Infusion | Scopolamine and  mecamylamine | 0.4 and 0.2 up to 15 mg | *There was a trend toward increased impairment in explicit memory for the mecamylamine + scopolamine condition as compared to scopolamine alone.* *Increased impairment was also seen for the mecamylamine 1 scopolamine condition as compared to scopolamine alone in selected behavioral ratings.* |
| Mewaldt 1979  [77] | Double-blind crossover RCT | Volunteers | 70 | 22.6  (18-32) | 35 | Immediate recall, Delayed recall, Recognition |  | intramuscular | scopolamine | 8 μg/kg | *Scopolamine did not affect recall of information learned prior to injection. Scopolamine did, however, impair performance in both the digit recall task and in the second series of memory tests.* |
| Mintzer 2001  [78] | double-blind crossover RCT | Volunteers | 19 | 32  (21-51) | 12 | Deese–Roediger–McDermott paradigm | 1 hour | Subcutaneous | Scopolamine | 0.3, 0.6 mg/70 kg | *Scopolamine produced dose-related reductions in both true and false recognition rates, and induced a more conservative response bias relative to placebo for recollection-based remember responses to studied words.* |
| Mintzer 2003  [79] | double-blind RCT | Volunteers | 48 | scopolamine group: 30.6±2.5 (SEM)  control group: 30.6±2.4 (SEM) | 20 | digit symbol substitution test, Two-Back Task, Recognition memory | - | subcutaneous | Scopolamine | 0.6 mg/70 kg | *At the studied doses scopolamine produced similar decrements in psychomotor performance, free recall, and overall sensitivity in distinguishing between studied and no studied items on a recognition memory test. However, the drugs differed with respect to effects on working memory, response bias, metacognition, subjective awareness, and selective attention.* |
| Mintzer 2005  [80] | double-blind RCT | Volunteers | 60 | Control group: 30.5±2.8  Scopolamine 0.3 and 0.6 groups: 25.6±2.4  27.9±2.4 | 31 | Circular lights task, cued recall test  Time of judgment of learning, | 55 min | subcutaneous | Scopolamine | 0.3, 0.6 mg/70 kg | *Results suggest that the drugs selectively impair those aspects of metamnemonic monitoring that require participants’ awareness of their overall current state of functioning (absolute accuracy of prospective item-by-item monitoring, prospective global monitoring) but not those that rely solely on assessment of individual item characteristics (relative accuracy of item-by-item monitoring).* |
| Mintzer 2007  [81] | double-blind crossover RCT | Volunteers | 20 | 27  (19-43) | 9 | Circular lights task, Manipulation and maintenance Task for working memory, | 55 min | subcutaneous | Scopolamine | 0.25, 0.5 mg/70 kg | *The effect of memory load on working memory is not affected by Scopolamine, at least within the limited range (5 and 7 letters) tested in this study.* |
| Mintzer 2010  [82] | double-blind RCT | Volunteers | 80 | Control group: 25.6±1.9 (SEM)  Scopolamine 0.25 and 0.5 groups: 28.5±2.2 (SEM)  25.3±2.1 (SEM) | 45 | Circular lights task, Cued recall test, | 50 min | subcutaneous | Scopolamine | 0.25, 0.5 mg/70 kg | *scopolamine impaired episodic memory (quantity and accuracy) but not semantic memory.* |
| Nakra 1992  [83] | Double blind RCT | Volunteers | 20 | 65.9±4.6 | 11 | Wechsler Memory Scale, Russell Adaptation Procedure | 90 min | Oral | trihexyphenidyl | 2mg | *Results indicated that the single 2-mg dose of trihexyphenidyl produced impaired performance on measures of immediate and half-hour delayed recall of complex verbal and visual material when compared to the placebo condition. However, differences were not found on several other memory measures, including general orientation, attention concentration, and learning of word associations.* |
| Newhouse 1994  [84] | Double blind RCT | Volunteers | 12 | 23.9±5 | 12 | Repeated Acquisition Test, High-Low Imagery Task, Selective Reminding, Choice-Reaction Time, High-Low Imagery Task | 120 min | Intravenous | mecamylamine | 5, 10, 20 mg | *the 20-mg dose caused a significant increase in errors in the learning condition of the Repeated Acquisition Task, producing a slower acquisition curve. There was no effect of drug on the performance component (retrieval of previously learned information). Reaction-time measures suggested a dose-related slowing of reaction time on several tasks.* |
| Newhouse 1992  [85] | Double blind crossover CT | Volunteers | 12 | 23.9±5 | 12 | Repeated acquisition test, Recognition memory, Selective Reminding task, Psychomotor ability | 120 min | Intravenous | mecamylamine | 5, 10, 20 mg | *20 mg dose caused a significant increase in errors in the learning condition of the Repeated Acquisition task, producing a slower acquisition curve. The lower doses produced less errors, but more than in the placebo condition. There was no effect of drug on the performance component (retrieval of previously learned information). On the recognition memory task, dose-related increases in false-alarms during the delay period were seen, with little effect on misses or hits. Reaction time measures suggested a dose-related slowing of RT on several tasks.* |
| Nuotto 1983  [86] | Double-blind crossover CT | Volunteers | 58 | Study I:  22.8±2.6  study II:  21.4±1.7  study III:  21.4±4.3 | 58 | simple reaction tests, Memory and learning task (digits) | 5-95 min | Intravenous | Scopolamine HBr | 0.3, 0.9  or 0.9 mg b.d. for 3 days | *Oral scopolamine hydrobromide in single doses of 0.3 mg and 0.9 mg, or 0.9 mg b.d. for 3 days, had few cognitive effects. A slight impairment of short-term memory and a decrease in the flicker fusion threshold were seen. In sufficient doses scopolamine impairs various psychomotor and cognitive skills.* |
| Obonsawin 1996  [87] | CT | Volunteers | 4 | -  (29-44) | 1 | continuous memory and recall tasks | - | Intravenous | Scopolamine | 0.5 mg | *Scopolamine altered performance on the recall task such that subjects were just as likely to recall words presented once as they were to recall words presented three times.* |
| Parrott 1985  [88] | double blind crossover CT | Volunteers | 38 | -  (18-40) | 37 | Choice reaction time Recognition component, letter cancellation, code substitution | 24 hours | Transdermal | Scopolamine | 0.5 mg | *Choice reaction time and code substitution performance levels were not significantly changed, but letter cancellation errors were significantly more frequent following transdermal scopolamine. Responses to the transdermal scopolamine patch seem to be quite variable.* |
| Parrott 1986  [89] | double-blind crossover CT | Volunteers | 12 | 24  (19-38) | 12 | Target tracking, Rapid visual information processing, Memory storage, letter cancellation | 1-2 hours | Oral and Transdermal | Scopolamine | 0.15, 0.3, 0.6, 1.2 mg | *Both transdermal and oral scopolamine produced significant linear dose-related decrements on tasks involving continuous attention, continuous performance, memory storage for new information, and on self-rated feelings of alertness and sociability.* |
| Parrott 1988  [90] | double-blind crossover CT | Volunteers | 28 | -  (18-40) | 28 | Letter cancellation, code substitution | 24 hours | Transdermal | Scopolamine |  | *Code substitution task performance was not affected, but letter cancellation errors were significantly increased, indicating an impairment in sustained attention with transdermal scopolamine.* |
| Parrott 1990  [91] | double-blind crossover CT | Volunteers | 12 | 21  (20-26) | 12 | A battery of psychological performance tests | 1-2,  5-6,  25-26, 29-30 Hours | Oral | scopolamine | 0.6  mg, t.i.d. | *Scopolamine impaired memory error and four choice reaction time.* |
| Paule 2004  [92] | double-blind crossover CT | Volunteers | 67 | 26.6±4.8 | 43 | Delayed matching to sample, Choice response latency | 45 min | Oral | Scopolamine | 0.4 mg | *Scopolamine alone decreased accuracy and with spinning, slowed speed.* |
| Petersen 1977  experiment 1 | RCT | Volunteers | 24 | 22.9±2.96 | 24 | Immediate and delayed recall, Acquisition, Delayed recall | 1-2 hours | Intravenous | Scopolamine | 5, 8, 10 µg/kg | *scopolamine has its primary effect on the acquisition of new material and less of an effect on the retrieval of information already learned. All three groups appeared to forget about the same amount of material over the 24 h recall period.* |
| Petersen 1977  experiment 2 [93] | RCT | Volunteers | 18 | 21.28±3.39 | 18 | Acquisition, Delayed recall | 1-2 hours | Intravenous | Scopolamine | 5, 10 µg/kg |  |
| Petersen 1979  [94] | RCT | Volunteers | 28 | 20.93±2.34 | 28 | Context cued recall, Free recall, Category recall task with and without cues | 45 min | Intravenous | Scopolamine | 2*5 µg/kg | *Two recall tasks which did not involve recall cues or prompts but not for the tasks involving memory acids. This implies that the drug state has memory cueing properties of its own and that recall can be enhanced either by restoring the drug state which existed at the time of learning or by providing external prompts.* |
| Pomara 2004  [95] | double-blind crossover RCT | Cognitively intact elderly adult | 24 | APOEε4 group: 67.33±4.58  APOEε4 negative group:  66.92±3.29 | 11 | Buschke Selective Reminding Task (BSRT) total and delayed recall | 1, 2.5, 5 hours | Oral | trihexyphenidyl | 1, 2 mg | *An acute administration of 2 mg of trihexyphenidyl produces significant impairments in total recall in both the e4 and the non-e4 participants. Participants with the e4 allele demonstrated significant impairments in delayed recall after both 1 and 2 mg doses of trihexyphenidyl while the non-e4 group did not.* |
| Pomara 2008  [96] | double-blind crossover RCT | Cognitively intact elderly adult | 24 | APOEε4 group: 67.33±4.58  APOEε4  negative group:  66.92±3.29 | 11 | Buschke Selective Reminding Task (BSRT) total and delayed recall | 1, 2.5, 5 hours | Oral | trihexyphenidyl | 1, 2 mg | *trihexyphenidyl produced increased ratings of mental slowness relative to placebo and that this effect was modulated by the APOE 4 allele* |
| Pomara 2010  [97] | double-blind crossover CT | Cognitively intact elderly adult | 24 | -  (62-76) | 11 | Word recall |  | Oral | trihexyphenidyl | 1, 2 mg | *Words studied before drug administration were better recalled following 2 mg trihexyphenidyl compared to placebo, and this RF effect was not affected by the APOE ε4 allele.* |
| Pompeia 2002  [98] | double-blind CT | Volunteers | 36 | 24.5±5.1 | 14 | Phonemic fluency, mean frequency of word produced in the easy category fluency task | 2 hours | Oral | Scopolamine | 0.6, 1.2 mg | *Low doses of scopolamine increased phonemic fluency. Semantic fluency was not increased by Scopolamine, although subjects treated with 1.2 mg of Scopolamine generated higher‐frequency words. Scopolamine did not affect clustering or switching.* |
| Potamianos 1982  [99] | Double blind CT | Volunteers | 13 | 82.9  (75-92) | 1 | Delayed recall, short story, digit span, orientation test | 90 min | Oral | Benzhexol | 2 mg | *All tests were significantly impaired by the benzhexol except for digit span.* |
| Potter 1992  [100] | crossover CT | Volunteers | 12 | - | 4 | Questionnaire, RT, accuracy of new/old words, signal detection | 30 min | Intravenous | Scopolamine | 0.4 mg | *Scopolamine caused a substantial impairment in task performance, but did not reduce the size of these old word/new word event-related potentials differences.* |
| Preston 1988  [101] | crossover CT | Volunteers | 20 | -  (19-41) | 10 | free recall, Sematic memory, Critical flicker fusion threshold, Sustained attention, Visual contrast sensitivity, Visual acuity, Total RT | - | Intramuscular | Scopolamine  Glycopyrrolate | 0.2, 0.4 mg 0.2 mg | *In the cognitive study, we found that scopolamine impaired subjects’ performance on verbal learning, spatial learning and choice reaction time. These changes were associated with subjective sedation as measured by analogue rating scales.* |
| Rabey 1996  [102] | Double blind RCT | Alzheimer's Disease, Parkinson’s Disease multiinfarct dementia, Volunteers | 9 | 67±5 | 4 | short Mental Test, Wechsler memory scale, logical memory, visual reproduction, learning. Digit span | - | Intravenous | Scopolamine | 0.5mg | *Scopolamine affected all the groups similarly, except for the Wechsler subtest of logic memory which showed larger deterioration in healthy controls compared to demented patients.* |
| Ramsdell 1996  [103] | double blind Crossover RCT | COPD | 20 | 73±6 | 8 | Visual search test, Paced Auditory Serial Addition Test, Wechsler Memory Scale, Benton Supraspan, California Verbal Learning Test, Fluency | - | - | Ipratropium | 2 puffs (180 g) qid  for 2 weeks | *There was no difference in performance scores on the cognitive tests among the three treatment periods.* *Significant cognitive impairment in the elderly is not commonly associated with treatment with either theophylline or ipratropium.* |
| Rasch 2006  [104] | double-blind crossover CT | Volunteers | 18 | 24.7  (21-29) | 18 | Word pair association task | 1 Hour | Intravenous  Oral | Scopolamine, mecamylamine | 4 µg/kg, 5 mg | *Compared to placebo, combined muscarinic and nicotinic receptor blockade significantly improved consolidation of declarative memories tested 10 hour later, but simultaneously impaired acquisition of similar material.* |
| Rasmusson 1979  [105] | CT | - | - | - | - | Maze learning, Map drawings, | 1 Hour | Oral | Scopolamine, methyl scopolamine | 2 mg, 1 mg | *A low oral dose of scopolamine which did not interfere with storage of a long list of digits interfered with some factors in a spatial learning task.* |
| Richardson 1984  [106] | crossover RCT | Volunteers | 16 | -  (20-52) | 12 | affirmative judgement, Mean confidence rating  Target list | 25min | Intravenous | Scopolamine | 5.7 µg/kg | *Scopolamine completely eliminated the subjects' ability to discriminate between successive lists of words in a test of recognition memory. Moreover, it also impaired the subjects' ability to reject homophones or synonyms of presented words. Thus, hyoscine appeared to impair the use of list tags, phonemic coding, and semantic coding in storing individual stimulus items in long-term memory.* |
| Richardson 1985  [107] | RCT | Volunteers | 30 | -  (>60) | - | MMSE, Symbol digit, Auditory verbal learning | 45min | Intramuscular | Scopolamine | 0.005mg/kg | *The very low dose of scopolamine resulted in a significant impairment of short-term memory but had no significant effect on global scores on the Mini Mental State nor on the Delirium Check List.* |
| Robbins 1997  [108] | CT | Volunteers | 24 | 29±6.3 | 24 | Pattern recognition, Matching to Sample (CANTAB) | 90min | - | Scopolamine | 200,  400,  600 µg | *Scopolamine significantly impaired accuracy of performance on a delayed matching to sample test of visual recognition memory in a dose- and delay dependent manner, but had only marginal decremental effects on a test of visuospatial paired associates learning. Scopolamine significantly lengthened decision times in a visual search matching to sample task at the 400 and 600 µg doses, without significantly affecting accuracy. The drug also impaired performance on tests of spatial (on accuracy and response time measures) and pattern (on response time only) memory.* |
| Roh 2013  [109] | double-blind Crossover RCT | Volunteers | 41 | 37±16 | 17 | continuous performance test-identical pairs, Stroop n-back task, visual spatial  working memory, hit RT variability | - | Oral | Mecamylamine | 10mg | *Mecamylamine worsened performance on continuous performance test-identical pairs, hit reaction time variability and a measure of attention.* |
| Rosier 1998  [110] | RCT | Volunteers | 24 | -  (19-24) | 10 | long-term visual recognition task | 70min | Oral | Scopolamine | 0.4, 0.8 mg | *Administration of scopolamine induced a significant deficit in delayed recognition performance. In contrast, a scopolamine challenge on delayed recognition following a drug-free encoding did not influence memory performance. In contrast, even at peak levels, scopolamine did not alter immediate recognition, detection or visual discriminative performances. Hence, the presence of scopolamine during the encoding of the shapes induced a significant long-term memory deficit that persisted after scopolamine clearance.* |
| Rusted 1988a  [111] | Double-blind Crossover CT | Volunteers | 20 | -  (18-29) | 20 | Digit span, Mental rotation, Mental reaction, Word lists task | 2 Hours | Oral | Scopolamine | 1.2mg | *Neither task was affected by scopolamine when completed alone or in combination with a secondary task. A concurrent secondary task reduced immediate free recall in a nonspecific fashion (i.e., spatial tapping or articulatory suppression impaired performance equally). Scopolamine significantly reduced the number of words recalled under all conditions* |
| Rusted 1988b  [112] | Double-blind Crossover CT | Volunteers | 20 | -  (18-29) | 20 | Baddeley logic task, Shape recognition task, Spatial and Visuo-spatial location Memory | 2 hours and 20 min | Subcutaneous | Scopolamine | 0.6mg | *Under standard presentation conditions, scopolamine significantly impaired performance on the problem-solving task and on tasks of visuo-spatial and spatial memory; memory for abstract shapes was not impaired. The results suggest that scopolamine impairs working memory, and that the decrement is at the level of the central executive mechanism rather than the subsystems which it controls.* |
| Rusted 1989  [113] | Double-blind crossover CT | Volunteers | 20 | -  (18-29) | 20 | recall of the word list learned | - | Subcutaneous | Scopolamine | 0.6mg | *While the placebo group achieved criterion within 4 acquisition trials, the group treated with scopolamine failed to reach criterion at all, with recall levelling off within 4 acquisition trials. Acquisition curves for subjects treated with scopolamine paralleled those obtained for the placebo group. Performance on delayed recall and recognition tests indicated that the subjects had in fact successfully encoded items in long-term memory, but had been unable to retrieve them in free recall.* |
| Rusted 1991  [114] | Double-blind crossover RCT | Volunteers | 18 | -  (18-30) | 18 | Digit span, Mental rotation, Free recall, Logic task | 90 min | - | Scopolamine | 1.2mg | *The results indicate that this model also fails to discriminate between the drug models (scopolamine and diazepam); both compounds selectively impaired tasks associated with the central executive mechanism and failed to disrupt tasks associated with the articulatory loop or the visuospatial scratchpad.* |
| Sambeth 2015  [115] | double-blind crossover CT | Volunteers | 16 | 23.4±3.2 | 7 | Verbal learning task, Continuous recognition memory test, Spatial memory task, Choice reaction time test, recognition trial | 1 hour | Oral | Biperiden | 2mg | *Biperiden impaired memory performance in the verbal learning task, the continuous recognition memory test, and the spatial memory task. Effects on attention and side effects, as measured using the choice reaction time test and questionnaires respectively, could be neglected.* |
| Schifano 1994  [116] | Double blind RCT | Volunteers | 36 | 27  (21-49) | 24 | Word-stem completion task, Word recall task, Word valence rating | 100-110 min | intramuscular | Scopolamine | 0.3, 0.6 mg | *Word recall showed some impairment following all active treatments.* |
| Schmedtje 1988  [117] | Double blind crossover RCT | Volunteers | 8 | - | - | Symbol digit, Simple reaction time, Pattern recognition | 150-180 min | Oral | Scopolamine | 0.4 mg | *No significant decrements were observed with the operational-level combined dose used in this study (0.4 mg oral scopolamine)* |
| Sherman 2003  [118] | CT | Volunteers | 16 | 22±0.6 | 2 | Episodic Memory and Recognition | - | - | Scopolamine | 0.4 mg | *Results showed that scopolamine affected responses to studied items, but not unstudied lures, demonstrating an unambiguous effect of scopolamine on recognition memory.* |
| Simmons 2008  [119] | double-blind RCT | Volunteers | 54 | 23.4±2.7 | 50 | ARES®  cognitive battery, Visual Accommodation | 120,  165 min | Intranasal (0.4mg) Oral (0.8 mg) | Scopolamine | 0.4,  0.8mg | *no significant treatment effects were detected over time regarding performance on the four ARES cognitive tasks in none of two groups.* |
| Simmons 2009  [120] | Crossover RCT | Volunteers | 16 | 23.5±2.96 | 13 | ARES®  cognitive battery, Code substitution task | 90 min | Intranasal | Scopolamine | 0.4mg | *The analyses revealed no significant cognitive performance effects over time between the placebo and treatment condition for the ARES® battery or for the code substitution test (delayed recall) included from the ANAM® battery.* |
| Simmons 2010  [121] | double-blind crossover RCT | Volunteers | 16 | 23.5±3 | 13 | Mean RT for delayed retrieval task | 195 min | Intranasal | Scopolamine | 0.4mg | *No significant effects on cognitive performance were found between the treatment and placebo conditions for the cognitive assessment data.* |
| Sommer 2005  [122] | CT | diurnal incontinence | 25 | 7.2±1.8 | 11 | Buschke style word list task, Recall, Digit span, Digit symbol | - | Oral | Oxybutynin | 2.5-5 mg for 4 weeks | *No significant impact of treatment type, indicating that oxybutynin did not impact cognition any more than behavior modification alone.* |
| Sperling 2002  [123] | double-blind crossover RCT | Volunteers | 10 | -  (23-35) | 10 | Recognition, RT, Free recall | - | Intravenous | Scopolamine | 0.4 mg | *Scopolamine, impaired performance on memory measures.* |
| Staskin 2010  [124] | CT | OAB | 14 | 68  (65-74) | 5 | HVLT-R | - | Oral | Trospium | 60 mg once daily over a 10-day | *Repeat memory testing revealed no significant net drug effect on learning or recall.* |
| Sunderland 1987  [125] | double-blind RCT | Alzheimer’s disease and Volunteers | 10 | 61.3±11.2 | 3 | Cognitive task, Digit span, Pfeiffer Short Portable Mental Status Questionnaire | 90 min | Intra venous | scopolamine | 0.1,  0.25,  0.5 mg | *Cognitive tests of new learning and semantic knowledge of the control population were essentially unchanged.* |
| Tariot 1996  [126] | double-blind crossover RCT | Volunteers | 23 | 63.2 | 8 | Category retrieval task words, Selective reminding test, RT, Trail making, Digit span | 90 min | Intravenous | Scopolamine HBr | 0.1,  0.25,  0.5 mg | *Scopolamine produced the expected dose-dependent impairments in most of the cognitive functions assessed. Behavioral and physiological measures were also affected, but only minimally.* |
| Thompson 2000  [127] | double-blind crossover CT | Volunteers | 6 | 22±2.1 | 6 | Inspection time | 150 min | Oral | Mecamylamine | 20mg | *There was a significant slowing of inspection time in the mecamylamine condition, compared to placebo.* |
| Tröster 1989  [128] | CT | Volunteers | 13 | 39.2  (31-59) | 13 | Free recall, Remote-memory battery, Fargo map test, New map test, Verbal-fluency, Brown-Peterson | - | Intramuscular | scopolamine HBr | 0.5, 0.8 mg | *Dose-related impairments on measures of anterograde short-term memory and verbal and nonverbal learning were observed and, in addition, performance on a category-fluency task was disrupted. In contrast, measures of remote memory were not affected by scopolamine treatment.* |
| Vitiello 1997  [129] | double-blind crossover RCT | Volunteers | 12 | 25±4 | 12 | RT, Word list learning, Fragmented picture test, Brown-Peterson, | 30 min | Intravenous | Scopolamine | 0.5mg | *Scopolamine-induced sedation, slowed information processing and impaired new learning and memory, but did not affect attention or retrieval from semantic memory.* |
| Voss 2010  [130] | Single blind cross-over CT | Volunteers | 15 | 25.57±2.2 | 15 | auditory verbal learning and memory test, Trail Making, Digit span, verbal fluency, Tower of London test | 90 min | Intravenous (Scopolamine), oral (Mecamylamine) | Scopolamine, Mecamylamine | 0.4 and 0.2 mg | *Results show that scopolamine significantly impaired the free recall and recognition performance in the verbal learning test. No other cognitive domain was affected, neither by scopolamine nor by mecamylamine.* |
| Wagg 2013  [131] | double-blind RCT | OAB (MMSE 20 or greater) | 794 | Fesoterodine group: 72.6±5.8  Control group: 72.8±5.7 | 367 | MMSE | - | Oral | Fesoterodine | 4, 8 mg for 12 weeks | *No meaningful mean change from baseline to week 12 in MMSE score was observed in the Fesoterodine or placebo  group.* |
| Wesnes 1983  [132] | double-blind CT | Volunteers | 20 | - | 12 | Hit probability, False alarm probability, Response bias | 60 min | Oral | Scopolamine | 0.6,  1.2 mg | *Scopolamine impaired detection performance, as a result of highly lowering of stimulus sensitivity, but not response bias.* |
| Wesnes 1984a  [133] | double-blind CT | Volunteers | 12 | 18-21 | 6 | Hit probability, RT, Stroop testing | 70-85min | Oral | Scopolamine | 1.2mg | *Scopolamine 1.2 mg produced a decrement in correct detections on the rapid visual information processing task. Scopolamine also disrupted performance on the Stroop test.* |
| Wesnes 1984b  [134] | double-blind CT | Volunteers | 12 | - | 6 | Hit probability | 20-30 min | Oral | Scopolamine, Methscopolamine | 0.6,  1.2 mg  1.2 mg | *scopolamine 1.2 mg produced a marked decrement in correct detections.* |
| Wesnes 1988  [135] | double-blind CT | Volunteers | 18 | 21.1 | 18 | Immediate and Delayed recall task (Words correct), recognition task, RT, Number matching, visual information processing, d2 task, Logical reasoning task, Stroop test | 60 min | Subcutaneous | Scopolamine | 0.6 mg | *Scopolamine produced marked and significant decrements on all major aspects of performance from the battery. The drug lowered the efficiency of the detection and processing of information in tests of visual vigilance, rapid information processing, choice reaction, letter cancellation and logical reasoning. Memory was also impaired on tests of immediate recall, delayed recall, recognition and memory scanning.* |
| Wesnes 2009  [136] | double-blind RCT | Volunteers | 12 | 69.1  (65-76) | 6 | Attention, Working Memory, Episodic Memory, Speed of Memory | 4 Hours | - | Solifenacin, Oxybutynin IR | 10 mg | *There was no evidence from absolute mean values or changes from baseline to suggest that Solifenacin 10 mg impaired cognition or self-ratings of mood and alertness versus placebo. Oxybutynin was associated with statistically significant impairments in Power of Attention, Continuity of Attention, Quality of Working Memory and Self-rated Alertness at a time point corresponding with its probable C_max_.* |
| Wetherell 1980  [137] | double-blind, crossover RCT | Volunteers | 16 | 22  (19-29) | - | Digit recall, Associative memory, Free recall, | 60 min | Intramuscular | Atropine | 2 mg | *At 60 min after dosing, atropine treated subjects recalled fewer digits than they did before treatment or when treated with placebo. Both atropine- and placebo treated subjects recalled a same amount of 'same-order' digits; and in 'reverse-order' digits difference was not significant.* |
| Wezenberg 2005  [138] | double-blind, crossover RCT | Volunteers | 16 | 66.1±4.46 | 8 | memory task, N back task, Digit Recall, Symbol Digit, Verbal Memory, Maze learning, RT | 90-132 min | Oral | Biperiden | 2 mg | *Episodic memory was impaired by biperiden. Working memory was non-significantly impaired by biperiden. Motor learning as well as visuospatial processes were impaired by biperiden.* |

# References

1. Atri A, Sherman S, Norman KA, Kirchhoff BA, Nicolas MM, Greicius MD, et al. Blockade of central cholinergic receptors impairs new learning and increases proactive interference in a word paired-associate memory task. Behavioral neuroscience. 2004;118(1):223.

2. Baakman AC, Alvarez‐Jimenez R, Rissmann R, Klaassen ES, Stevens J, Goulooze SC, et al. An anti‐nicotinic cognitive challenge model using mecamylamine in comparison with the anti‐muscarinic cognitive challenge using scopolamine. British journal of clinical pharmacology. 2017;83(8):1676-87.

3. Beatty WW, Butters N, Janowsky DS. Patterns of memory failure after scopolamine treatment: implications for cholinergic hypotheses of dementia. Behavioral and Neural Biology. 1986;45(2):196-211.

4. Bishop KI, Curran, H. V., & Lader, M. . Do scopolamine and lorazepam have dissociable effects on human memory systems? A dose–response study with normal volunteers. . Experimental and Clinical Psychopharmacology, 4(3), 292–299. 1996.

5. Borghans LG, Blokland A, Sambeth A. Effects of biperiden and acute tryptophan depletion and their combination on verbal word memory and EEG. Psychopharmacology. 2017;234(7):1135-43.

6. Borghans L, Sambeth A, Blokland A. Biperiden Selectively Impairs Verbal Episodic Memory in a Dose-and Time-Dependent Manner in Healthy Subjects. Journal of Clinical Psychopharmacology. 2020;40(1):30-7.

7. Brandeis D, Naylor H, Halliday R, Callaway E, Yano L. Scopolamine effects on visual information processing, attention, and event‐related potential map latencies. Psychophysiology. 1992;29(3):315-35.

8. Broks P, Preston G, Traub M, Poppleton P, Ward C, Stahl S. Modelling dementia: effects of scopolamine on memory and attention. Neuropsychologia. 1988;26(5):685-700.

9. Brown SB, Van der Wee NJ, Van Noorden MS, Giltay EJ, Nieuwenhuis S. Noradrenergic and cholinergic modulation of late ERP responses to deviant stimuli. Psychophysiology. 2015;52(12):1620-31.

10. Brown SB, Slagter HA, Van Noorden MS, Giltay EJ, Van Der Wee NJ, Nieuwenhuis S. Effects of clonidine and scopolamine on multiple target detection in rapid serial visual presentation. Psychopharmacology. 2016;233(2):341-50.

11. Bukala BR, Browning M, Cowen PJ, Harmer CJ, Murphy SE. Overnight transdermal scopolamine patch administration has no clear effect on cognition and emotional processing in healthy volunteers. Journal of Psychopharmacology. 2019;33(2):255-7.

12. Caine ED, Weingartner H, Ludlow CL, Cudahy EA, Wehry S. Qualitative analysis of scopolamine-induced amnesia. Psychopharmacology. 1981;74(1):74-80.

13. Callaway E, Band RI. Some psychopharmacological effects of atropine; preliminary investigation of broadened attention. AMA Arch Neurol Psychiatry. 1958;79(1):91-102.

14. Callaway E, Halliday R, Naylor H, Schechter G. Effects of oral scopolamine on human stimulus evaluation. Psychopharmacology (Berl). 1985;85(2):133-8.

15. Chhatwal JP, Schultz AP, Hedden T, Boot BP, Wigman S, Rentz D, et al. Anticholinergic amnesia is mediated by alterations in human network connectivity architecture. Cerebral Cortex. 2019;29(8):3445-56.

16. Craig M, Brammer M, Maki P, Fletcher P, Daly E, Rymer J, et al. The interactive effect of acute ovarian suppression and the cholinergic system on visuospatial working memory in young women. Psychoneuroendocrinology. 2010;35(7):987-1000.

17. Crow T, Grove-White I, Kelman G. Differential effect of atropine and hyoscine on human learning capacity. British journal of pharmacology. 1971;43(2):464P.

18. Crow T, Grove-White I. An analysis of the learning deficit following hyoscine administration to man. British Journal of Pharmacology. 1973;49(2):322.

19. Crow T, Grove-White I, Ross D. The specificity of the action of hyoscine on human learning [proceedings]. British Journal of Clinical Pharmacology. 1975;2(4):367P-8P.

20. Curran HV, Schifano F, Lader M. Models of memory dysfunction? A comparison of the effects of scopolamine and lorazepam on memory, psychomotor performance and mood. Psychopharmacology. 1991;103(1):83-90.

21. Curran HV, Pooviboonsuk P, Dalton JA, Lader MH. Differentiating the effects of centrally acting drugs on arousal and memory: an event-related potential study of scopolamine, lorazepam and diphenhydramine. Psychopharmacology (Berl). 1998;135(1):27-36.

22. Danion JM, Zimmermann MA, Willard-Schroeder D, Grangé D, Welsch M, Imbs JL, et al. Effects of scopolamine, trimipramine and diazepam on explicit memory and repetition priming in healthy volunteers. Psychopharmacology (Berl). 1990;102(3):422-4.

23. Diefenbach K, Donath F, Maurer A, Bravo SQ, Wernecke K-D, Schwantes U, et al. Randomised, double-blind study of the effects of oxybutynin, tolterodine, trospium chloride and placebo on sleep in healthy young volunteers. Clinical drug investigation. 2003;23(6):395-404.

24. Diefenbach K, Arold G, Wollny A, Schwantes U, Haselmann J, Roots I. Effects on sleep of anticholinergics used for overactive bladder treatment in healthy volunteers aged > or = 50 years. BJU Int. 2005;95(3):346-9.

25. Drachman DA, Leavitt J. Human memory and the cholinergic system: a relationship to aging? Archives of neurology. 1974;30(2):113-21.

26. Dubeau CE, Kraus SR, Griebling TL, Newman DK, Wyman JF, Johnson TM, 2nd, et al. Effect of fesoterodine in vulnerable elderly subjects with urgency incontinence: a double-blind, placebo controlled trial. J Urol. 2014;191(2):395-404.

27. Duka T, Edelmann V, Schütt B, Dorow R, Fichte K. Scopolamine-induced amnesia in humans: lack of effects of the benzodiazepine receptor antagonist β-carboline ZK 93426. Journal of psychopharmacology. 1992;6(3):382-8.

28. Duka T, Redemann B, Voet B. Scopolamine and lorazepam exert different patterns of effects in a test battery assessing stages of information processing. Psychopharmacology. 1995;119(3):315-24.

29. Duka T, Ott H, Rohloff A, Voet B. The effects of a benzodiazepine receptor antagonist β-carboline ZK-93426 on scopolamine-induced impairment on attention, memory and psychomotor skills. Psychopharmacology. 1996;123(4):361-73.

30. Dundee JW, Pandit SK. Anterograde amnesic effects of pethidine, hyoscine and diazepam in adults. Br J Pharmacol. 1972;44(1):140-4.

31. Dunne MP, Hartley LR. The effects of scopolamine upon verbal memory: evidence for an attentional hypothesis. Acta Psychol (Amst). 1985;58(3):205-17.

32. Dunne MP, Hartley LR. Scopolamine and the control of attention in humans. Psychopharmacology (Berl). 1986;89(1):94-7.

33. Dunne MP. Scopolamine and sustained retrieval from semantic memory. J Psychopharmacol. 1990;4(1):13-8.

34. Dunne MP, Statham D, Raphael B, Kemp R, Kelly B. Further evidence that scopolamine can improve verbal fluency. J Psychopharmacol. 1993;7(2):159-63.

35. Ebert U, Siepmann M, Oertel R, Wesnes KA, Kirch W. Pharmacokinetics and pharmacodynamics of scopolamine after subcutaneous administration. The Journal of Clinical Pharmacology. 1998;38(8):720-6.

36. Edginton T, Rusted JM. Separate and combined effects of scopolamine and nicotine on retrieval-induced forgetting. Psychopharmacology (Berl). 2003;170(4):351-7.

37. Ellis JR, Ellis KA, Bartholomeusz CF, Harrison BJ, Wesnes KA, Erskine FF, et al. Muscarinic and nicotinic receptors synergistically modulate working memory and attention in humans. Int J Neuropsychopharmacol. 2006;9(2):175-89.

38. Erskine FF, Ellis J, Ellis KA, Stuber E, Hogan K, Miller V, et al. Evidence for synergistic modulation of early information processing by nicotinic and muscarinic receptors in humans. Human Psychopharmacology: Clinical and Experimental. 2004;19(7):503-9.

39. Flicker C, Serby M, Ferris SH. Scopolamine effects on memory, language, visuospatial praxis and psychomotor speed. Psychopharmacology (Berl). 1990;100(2):243-50.

40. Flicker C, Ferris SH, Serby M. Hypersensitivity to scopolamine in the elderly. Psychopharmacology. 1992;107(2-3):437-41.

41. Fredrickson A, Snyder PJ, Cromer J, Thomas E, Lewis M, Maruff P. The use of effect sizes to characterize the nature of cognitive change in psychopharmacological studies: an example with scopolamine. Human Psychopharmacology: Clinical and Experimental. 2008;23(5):425-36.

42. Frith C, Richardson J, Samuel M, Crow T, McKenna P. The effects of intravenous diazepam and hyoscine upon human memory. The Quarterly Journal of Experimental Psychology Section A. 1984;36(1):133-44.

43. Frith C, McGinty M, Gergel I, Crow T. The effects of scopolamine and clonidine upon the performance and learning of a motor skill. Psychopharmacology. 1989;98(1):120-5.

44. Geller EJ, Dumond JB, Bowling JM, Khandelwal CM, Wu JM, Busby-Whitehead J, et al. Effect of trospium chloride on cognitive function in women aged 50 and older: a randomized trial. Female pelvic medicine & reconstructive surgery. 2017;23(2):118-23.

45. Ghoneim M, Mewaldt S. Effects of diazepam and scopolamine on storage, retrieval and organizational processes in memory. Psychopharmacologia. 1975;44(3):257-62.

46. Ghoneim MM, Mewaldt SP. Studies on human memory: the interactions of diazepam, scopolamine, and physostigmine. Psychopharmacology (Berl). 1977;52(1):1-6.

47. Giramonti KM, Kogan BA, Halpern LF. The effects of anticholinergic drugs on attention span and short‐term memory skills in children. Neurourology and Urodynamics: Official Journal of the International Continence Society. 2008;27(4):315-8.

48. Golding J, Strong R, Pethybridge R. Time-course of effects of oral cinnarizine and hyoscine on task performance. Journal of Psychopharmacology. 1989;3(4):187-97.

49. Golding JF, Wesnes KA, Leaker BR. The effects of the selective muscarinic M3 receptor antagonist darifenacin, and of hyoscine (scopolamine), on motion sickness, skin conductance & cognitive function. British journal of clinical pharmacology. 2018;84(7):1535-43.

50. Gordon C, Gonen A, Nachum Z, Doweck I, Spitzer O, Shupak A. The effects of dimenhydrinate, cinnarizine and transdermal scopolamine on performance. Journal of Psychopharmacology. 2001;15(3):167-72.

51. Grasby P, Frith C, Paulesu E, Friston K, Frackowiak R, Dolan RJ. The effect of the muscarinic antagonist scopolamine on regional cerebral blood flow during the performance of a memory task. Experimental brain research. 1995;104(2):337-48.

52. Green A, Ellis KA, Ellis J, Bartholomeusz CF, Ilic S, Croft RJ, et al. Muscarinic and nicotinic receptor modulation of object and spatial n-back working memory in humans. Pharmacology Biochemistry and Behavior. 2005;81(3):575-84.

53. Grober E, Leipzig RM, Lipton RB, Wisniewski W, Schroeder M, Davies P, et al. Does scopolamine directly impair memory? J Cogn Neurosci. 1989;1(4):327-35.

54. Harel BT, Pietrzak RH, Snyder PJ, Maruff P. Effect of cholinergic neurotransmission modulation on visual spatial paired associate learning in healthy human adults. Psychopharmacology. 2013;228(4):673-83.

55. Hardy T, Wakely D. The amnesic properties of hyoscine and atropine in pre‐anaesthetic medication. Anaesthesia. 1962;17(3):331-6.

56. Higgins ST, Woodward BM, Henningfield JE. Effects of atropine on the repeated acquisition and performance of response sequences in humans. Journal of the experimental analysis of behavior. 1989;51(1):5-15.

57. Hongyu X, Qingting W, Xiaoling S, Liwu Z, Ailing Y, Xin L. Penehyclidine hydrochloride on postoperatively cognitive function. Medical hypotheses. 2019;129:109246.

58. Howland J, Rohsenow DJ, Minsky S, Snoberg J, Tagerud S, Hunt SK, et al. The Effects of Transdermal Scopolamine on Simulated Ship Navigation and Attention/Reaction Time. International Journal of Occupational and Environmental Health. 2008;14(4):250-6.

59. Jacob Huff F, Mickel SF, Corkin S, Growdon JH. Cognitive functions affected by scopolamine in Alzheimer's disease and normal aging. Drug development research. 1988;12(3‐4):271-8.

60. Jones DM, Jones M, Lewis MJ, Spriggs T. Drugs and human memory: effects of low doses of nitrazepam and hyoscine on retention. British Journal of Clinical Pharmacology. 1979;7(5):479-83.

61. Kamboj S, Curran H. Scopolamine induces impairments in the recognition of human facial expressions of anger and disgust. Psychopharmacology. 2006;185(4):529-35.

62. Kamboj SK, Curran HV. Neutral and emotional episodic memory: global impairment after lorazepam or scopolamine. Psychopharmacology. 2006;188(4):482-8.

63. Katz IR, Sands LP, Bilker W, DiFilippo S, Boyce A, D'Angelo K. Identification of medications that cause cognitive impairment in older people: the case of oxybutynin chloride. Journal of the American Geriatrics Society. 1998;46(1):8-13.

64. Kay GG, Wesnes KA. Pharmacodynamic effects of darifenacin, a muscarinic M selective receptor antagonist for the treatment of overactive bladder, in healthy volunteers. BJU Int. 2005;96(7):1055-62.

65. Kay G, Crook T, Rekeda L, Lima R, Ebinger U, Arguinzoniz M, et al. Differential effects of the antimuscarinic agents darifenacin and oxybutynin ER on memory in older subjects. Eur Urol. 2006;50(2):317-26.

66. Kay GG, Maruff P, Scholfield D, Malhotra B, Whelan L, Darekar A, et al. Evaluation of cognitive function in healthy older subjects treated with fesoterodine. Postgrad Med. 2012;124(3):7-15.

67. Kay GG, Staskin DR, MacDiarmid S, McIlwain M, Dahl NV. Cognitive Effects of Oxybutynin Chloride Topical Gel in Older Healthy Subjects. Clinical drug investigation. 2012;32(10):707-14.

68. Klinkenberg I, Blokland A, Riedel W, Sambeth A. Human electrophysiological correlates of learned irrelevance: effects of the muscarinic M1 antagonist biperiden. International Journal of Neuropsychopharmacology. 2012;15(10):1375-85.

69. Koller G, Satzger W, Adam M, Wagner M, Kathmann N, Soyka M, et al. Effects of scopolamine on matching to sample paradigm and related tests in human subjects. Neuropsychobiology. 2003;48(2):87-94.

70. Kosilov K, Kuzina I, Loparev S, Gainullina Y, Kosilova L, Prokofyeva A. Influence of the short-term intake of high doses of solifenacin and trospium on cognitive function and health-related quality of life in older women with urinary incontinence. International neurourology journal. 2018;22(1):41.

71. Lavoie KL, Sedeno M, Hamilton A, Li P-Z, De Sousa D, Troosters T, et al. Behavioural interventions targeting physical activity improve psychocognitive outcomes in COPD. ERJ open research. 2019;5(4).

72. Liem‐Moolenaar M, de Boer P, Timmers M, Schoemaker RC, van Hasselt JC, Schmidt S, et al. Pharmacokinetic–pharmacodynamic relationships of central nervous system effects of scopolamine in healthy subjects. British journal of clinical pharmacology. 2011;71(6):886-98.

73. Lines C, Preston G, Broks P, Dawson C. The effects of scopolamine on retrieval from semantic memory. Journal of Psychopharmacology. 1991;5(3):234-7.

74. Lipton RB, Kolodner K, Wesnes K. Assessment of cognitive function of the elderly population: effects of darifenacin. The Journal of urology. 2005;173(2):493-8.

75. Little J, Broocks A, Martin A, Hill J, Tune L, Mack C, et al. Serotonergic modulation of anticholinergic effects on cognition and behavior in elderly humans. Psychopharmacology. 1995;120(3):280-8.

76. Little JT, Johnson DN, Minichiello M, Weingartner H, Sunderland T. Combined nicotinic and muscarinic blockade in elderly normal volunteers: cognitive, behavioral, and physiologic responses. Neuropsychopharmacology. 1998;19(1):60-9.

77. Mewaldt S, Ghoneim M. The effects and interactions of scopolamine, physostigmine and methamphetamine on human memory. Pharmacology Biochemistry and Behavior. 1979;10(2):205-10.

78. Mintzer MZ, Griffiths RR. Acute dose-effects of scopolamine on false recognition. Psychopharmacology. 2001;153(4):425-33.

79. Mintzer MZ, Griffiths RR. Lorazepam and scopolamine: A single-dose comparison of effects on human memory and attentional processes. Experimental and Clinical Psychopharmacology. 2003;11(1):56.

80. Mintzer MZ, Griffiths RR. Drugs, memory, and metamemory: A dose-effect study with lorazepam and scopolamine. Experimental and Clinical Psychopharmacology. 2005;13(4):336.

81. Mintzer MZ, Griffiths RR. Differential effects of scopolamine and lorazepam on working memory maintenance versus manipulation processes. Cognitive, Affective, & Behavioral Neuroscience. 2007;7(2):120-9.

82. Mintzer MZ, Kleykamp BA, Griffiths RR. Dose effects of triazolam and scopolamine on metamemory. Experimental and clinical psychopharmacology. 2010;18(1):17.

83. Nakra BR, Margolis RB, Gfeller JD, Grossberg GT, Sata LS. The effect of a single low dose of trihexyphenidyl on memory functioning in the healthy elderly. Int Psychogeriatr. 1992;4(2):207-14.

84. Newhouse PA, Potter A, Corwin J, Lenox R. Age-related effects of the nicotinic antagonist mecamylamine on cognition and behavior. Neuropsychopharmacology. 1994;10(2):93-107.

85. Newhouse PA, Potter A, Corwin J, Lenox R. Acute nicotinic blockade produces cognitive impairment in normal humans. Psychopharmacology (Berl). 1992;108(4):480-4.

86. Nuotto E. Psychomotor, physiological and cognitive effects of scopolamine and ephedrine in healthy man. European journal of clinical pharmacology. 1983;24(5):603-9.

87. Obonsawin MC, Goddard C, Crawford JR, Al‐Mousawi A, Evans NT, Roeda D, et al. The effects of scopolamine on the recall of repeated words: a preliminary investigation. Human Psychopharmacology: Clinical and Experimental. 1996;11(1):25-31.

88. Parrott A, Jones R. Effects of transdermal scopolamine upon psychological test performance at sea. European journal of clinical pharmacology. 1985;28(4):419-23.

89. Parrott AC. The effects of transdermal scopolamine and four dose levels of oral scopolamine (0.15, 0.3, 0.6, and 1.2 mg) upon psychological performance. Psychopharmacology. 1986;89(3):347-54.

90. Parrott A. Transdermal scopolamine: effects upon psychological performance and visual functioning at sea. Human Psychopharmacology: Clinical and Experimental. 1988;3(2):119-25.

91. Parrott A, Golding J, Pethybridge R. The effects of single and repeated doses of oral scopolamine, cinnarizine, and placebo upon psychological performance and physiological functioning. Human Psychopharmacology: Clinical and Experimental. 1990;5(3):207-16.

92. Paule MG, Chelonis JJ, Blake DJ, Dornhoffer JL. Effects of drug countermeasures for space motion sickness on working memory in humans. Neurotoxicology and teratology. 2004;26(6):825-37.

93. Petersen RC. Scopolamine induced learning failures in man. Psychopharmacology. 1977;52(3):283-9.

94. Petersen RC. Scopolamine state-dependent memory processes in man. Psychopharmacology. 1979;64(3):309-14.

95. Pomara N, Willoughby LM, Wesnes K, Sidtis JJ. Increased Anticholinergic Challenge-Induced Memory Impairment Associated with the APOE-ɛ 4 Allele in the Elderly: A Controlled Pilot Study. Neuropsychopharmacology. 2004;29(2):403-9.

96. Pomara N, Belzer K, Sidtis JJ, Hernando R, De La Pena C. Increased Mental Slowing Associated With the APOE ɛ4 Allele After Trihexyphenidyl Oral Anticholinergic Challenge in Healthy Elderly. The American Journal of Geriatric Psychiatry. 2008;16(2):116-24.

97. Pomara N, Yi L, Belzer K, Facelle TM, Willoughby LM, Sidtis JJ. Retrograde facilitation of verbal memory by trihexyphenidyl in healthy elderly with and without the APOE ε4 allele. European Neuropsychopharmacology. 2010;20(7):467-72.

98. Pompéia S, Rusted JM, Curran HV. Verbal fluency facilitated by the cholinergic blocker, scopolamine. Hum Psychopharmacol. 2002;17(1):51-9.

99. Potamianos G, Kellett J. Anti-cholinergic drugs and memory: the effects of benzhexol on memory in a group of geriatric patients. The British Journal of Psychiatry. 1982;140(5):470-2.

100. Potter DD, Pickles CD, Roberts RC, Rugg MD. The effects of scopolamine on event‐related potentials in a continuous recognition memory task. Psychophysiology. 1992;29(1):29-37.

101. Preston G, Brazell C, Ward C, Broks P, Traub M, Stahl S. The scopolamine model of dementia: determination of central cholinomimetic effects of physostigmine on cognition and biochemical markers in man. Journal of psychopharmacology. 1988;2(2):67-79.

102. Rabey J, Neufeld M, Treves T, Sifris P, Korczyn A. Cognitive effects of scopolamine in dementia. Journal of neural transmission. 1996;103(7):873-81.

103. Ramsdell JW, Henderson S, Renvall MJ, Salmon DP, Ferguson P. Effects of theophylline and ipratropium on cognition in elderly patients with chronic obstructive pulmonary disease. Annals of Allergy, Asthma & Immunology. 1996;76(4):335-40.

104. Rasch BH, Born J, Gais S. Combined blockade of cholinergic receptors shifts the brain from stimulus encoding to memory consolidation. Journal of cognitive neuroscience. 2006;18(5):793-802.

105. Rasmusson D, Dudar J. Effect of scopolamine on maze learning performance in humans. Experientia. 1979;35(8):1069-70.

106. Richardson J, Frith C, Scott E, Crow T, Cunningham-Owens D. The effects of intravenous diazepam and hyoscine upon recognition memory. Behavioural brain research. 1984;14(3):193-9.

107. Richardson JS, Miller PS, Lemay JS, Jyu CA, Neil SG, Kilduff CJ, et al. Mental dysfunction and the blockade of muscarinic receptors in the brains of the normal elderly. Progress in Neuro-Psychopharmacology and Biological Psychiatry. 1985;9(5-6):651-4.

108. Robbins T, Semple J, Kumar R, Truman M, Shorter J, Ferraro A, et al. Effects of scopolamine on delayed-matching-to-sample and paired associates tests of visual memory and learning in human subjects: comparison with diazepam and implications for dementia. Psychopharmacology. 1997;134(1):95-106.

109. Roh S, Hoeppner SS, Schoenfeld D, Fullerton CA, Stoeckel LE, Evins AE. Acute effects of mecamylamine and varenicline on cognitive performance in non-smokers with and without schizophrenia. Psychopharmacology (Berl). 2014;231(4):765-75.

110. Rosier A, Cornette L, Orban GA. Scopolamine-induced impairment of delayed recognition of abstract visual shapes. Neuropsychobiology. 1998;37(2):98-103.

111. Rusted JM. Dissociative effects of scopolamine on working memory in healthy young volunteers. Psychopharmacology (Berl). 1988;96(4):487-92.

112. Rusted JM, Warburton DM. The effects of scopolamine on working memory in healthy young volunteers. Psychopharmacology (Berl). 1988;96(2):145-52.

113. Rusted JM, Warburton DM. Effects of scopolamine on verbal memory; a retrieval or acquisition deficit? Neuropsychobiology. 1989;21(2):76-83.

114. Rusted JM, Eaton-Williams P, Warburton DM. A comparison of the effects of scopolamine and diazepam on working memory. Psychopharmacology (Berl). 1991;105(3):442-5.

115. Sambeth A, Riedel WJ, Klinkenberg I, Kähkönen S, Blokland A. Biperiden selectively induces memory impairment in healthy volunteers: no interaction with citalopram. Psychopharmacology (Berl). 2015;232(11):1887-97.

116. Schifano F, Curran HV. Pharmacological models of memory dysfunction? A comparison of the effects of scopolamine and lorazepam on word valence ratings, priming and recall. Psychopharmacology (Berl). 1994;115(3):430-4.

117. Schmedtje JF, Jr., Oman CM, Letz R, Baker EL. Effects of scopolamine and dextroamphetamine on human performance. Aviat Space Environ Med. 1988;59(5):407-10.

118. Sherman SJ, Atri A, Hasselmo ME, Stern CE, Howard MW. Scopolamine impairs human recognition memory: data and modeling. Behav Neurosci. 2003;117(3):526-39.

119. Simmons RG, Phillips JB, Lojewski RA, Lawson BD. A COMPARISON OF INTRANASAL AND ORAL SCOPOLAMINE FOR MOTION SICKNESS PREVENTION IN MILITARY PERSONNEL. NAVAL AEROSPACE MEDICAL RESEARCH LABORATORY 280 FRED BAUER STREET BUILDING 1811 PENSACOLA, FLORIDA 32508 NAMRL TECHNICAL REPORT 08-10. 2008.

120. Simmons RG, Phillips JB, Lojewski RA. EFFICACY OF INTRANASAL SCOPOLAMINE GEL FOR MOTION SICKNESS TREATMENT IN AVIATION CANDIDATES. NAVAL AEROSPACE MEDICAL RESEARCH LABORATORY 280 FRED BAUER STREET PENSACOLA NAS, FL 32508 NAMRL TECHNICAL REPORT 09-17. 2009.

121. Simmons RG, Phillips JB, Lojewski RA, Wang Z, Boyd JL, Putcha L. The efficacy of low-dose intranasal scopolamine for motion sickness. Aviat Space Environ Med. 2010;81(4):405-12.

122. Sommer BR, O'Hara R, Askari N, Kraemer HC, Kennedy WA, 2nd. The effect of oxybutynin treatment on cognition in children with diurnal incontinence. J Urol. 2005;173(6):2125-7.

123. Sperling R, Greve D, Dale A, Killiany R, Holmes J, Rosas HD, et al. Functional MRI detection of pharmacologically induced memory impairment. Proc Natl Acad Sci U S A. 2002;99(1):455-60.

124. Staskin D, Kay G, Tannenbaum C, Goldman HB, Bhashi K, Ling J, et al. Trospium chloride has no effect on memory testing and is assay undetectable in the central nervous system of older patients with overactive bladder. Int J Clin Pract. 2010;64(9):1294-300.

125. Sunderland T, Tariot PN, Cohen RM, Weingartner H, Mueller EA, 3rd, Murphy DL. Anticholinergic sensitivity in patients with dementia of the Alzheimer type and age-matched controls. A dose-response study. Arch Gen Psychiatry. 1987;44(5):418-26.

126. Tariot PN, Patel SV, Cox C, Henderson RE. Age-related decline in central cholinergic function demonstrated with scopolamine. Psychopharmacology (Berl). 1996;125(1):50-6.

127. Thompson JC, Stough C, Ames D, Ritchie C, Nathan PJ. Effects of the nicotinic antagonist mecamylamine on inspection time. Psychopharmacology (Berl). 2000;150(1):117-9.

128. Tröster AI, Beatty WW, Staton RD, Rorabaugh AG. Effects of scopolamine on anterograde and remote memory in humans. Psychobiology. 1989;17(1):12-8.

129. Vitiello B, Martin A, Hill J, Mack C, Molchan S, Martinez R, et al. Cognitive and behavioral effects of cholinergic, dopaminergic, and serotonergic blockade in humans. Neuropsychopharmacology. 1997;16(1):15-24.

130. Voss B, Thienel R, Reske M, Habel U, Kircher T. Cognitive performance and cholinergic transmission: influence of muscarinic and nicotinic receptor blockade. Eur Arch Psychiatry Clin Neurosci. 2010;260 Suppl 2:S106-10.

131. Wagg A, Khullar V, Marschall-Kehrel D, Michel MC, Oelke M, Darekar A, et al. Flexible-dose fesoterodine in elderly adults with overactive bladder: results of the randomized, double-blind, placebo-controlled study of fesoterodine in an aging population trial. J Am Geriatr Soc. 2013;61(2):185-93.

132. Wesnes K, Warburton DM. Effects of scopolamine on stimulus sensitivity and response bias in a visual vigilance task. Neuropsychobiology. 1983;9(2-3):154-7.

133. Wesnes K, Revell A. The separate and combined effects of scopolamine and nicotine on human information processing. Psychopharmacology (Berl). 1984;84(1):5-11.

134. Wesnes K, Warburton DM. Effects of scopolamine and nicotine on human rapid information processing performance. Psychopharmacology (Berl). 1984;82(3):147-50.

135. Wesnes K, Simpson P, Kidd A. An investigation of the range of cognitive impairments induced by scopolamine 0.6 mg s.c. Human Psychopharmacology: Clinical and Experimental. 1988;3(1):27-41.

136. Wesnes KA, Edgar C, Tretter RN, Bolodeoku J. Exploratory pilot study assessing the risk of cognitive impairment or sedation in the elderly following single doses of solifenacin 10 mg. Expert Opin Drug Saf. 2009;8(6):615-26.

137. Wetherell A. Some effects of atropine on short-term memory. British journal of clinical pharmacology. 1980;10(6):627-8.

138. Wezenberg E, Verkes RJ, Sabbe BG, Ruigt GS, Hulstijn W. Modulation of memory and visuospatial processes by biperiden and rivastigmine in elderly healthy subjects. Psychopharmacology (Berl). 2005;181(3):582-94.
