# Supplementary material for: Cognitive effects of individual anticholinergic drugs: a systematic review and meta-analysis
Source: Dement Neuropsychol. 2023 May 29;17:e20220053. doi: 10.1590/1980-5764-DN-2022-0053 (PMC10229087; doi:10.1590/1980-5764-DN-2022-0053)
Supplement: Supplementary file 3 [file 1980-5764-DN-17-e20220053-Suppl03.docx]

**Supplementary Material 3**

Forest plot of assessment of effects of scopolamine on digit span.


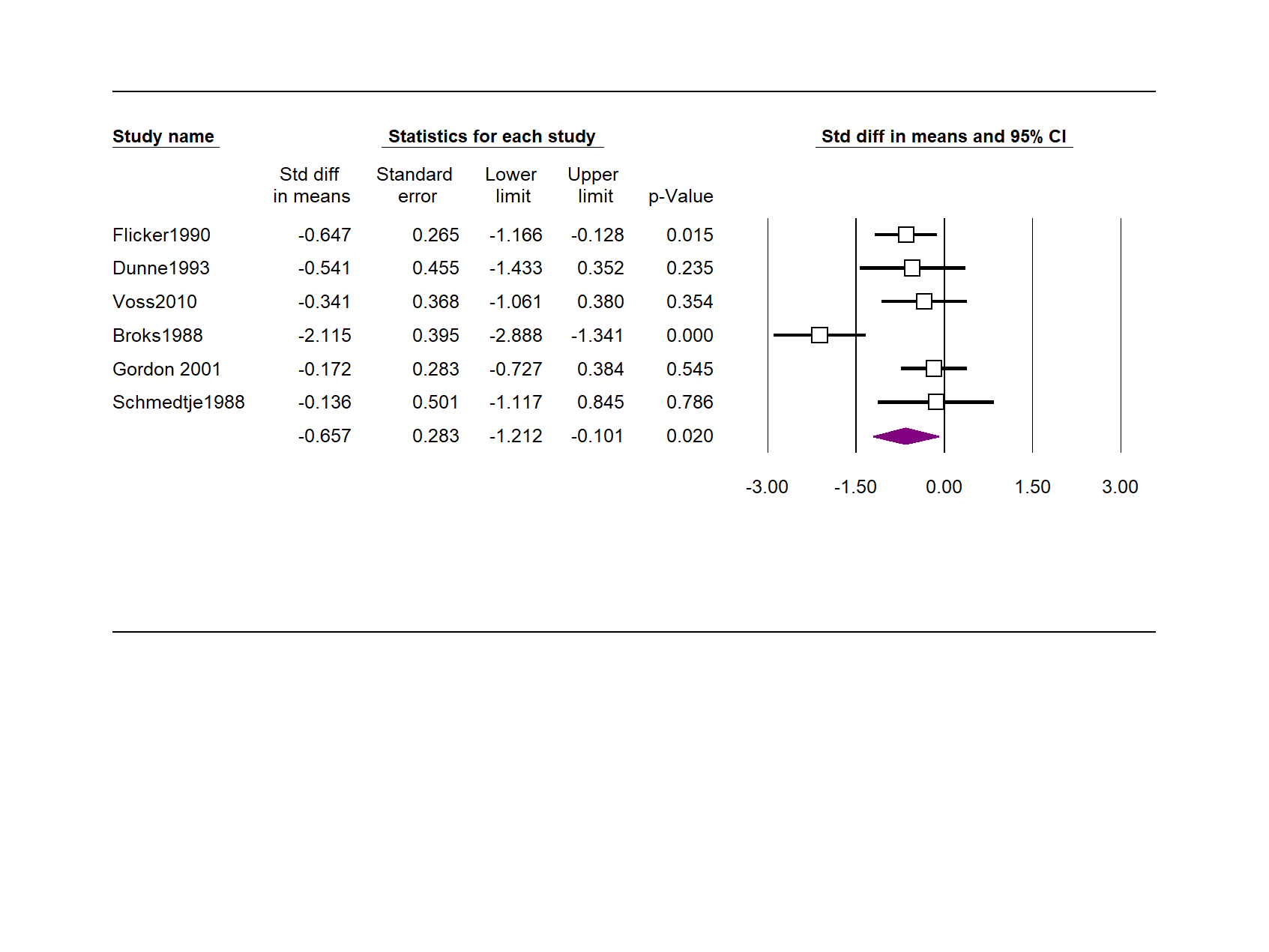


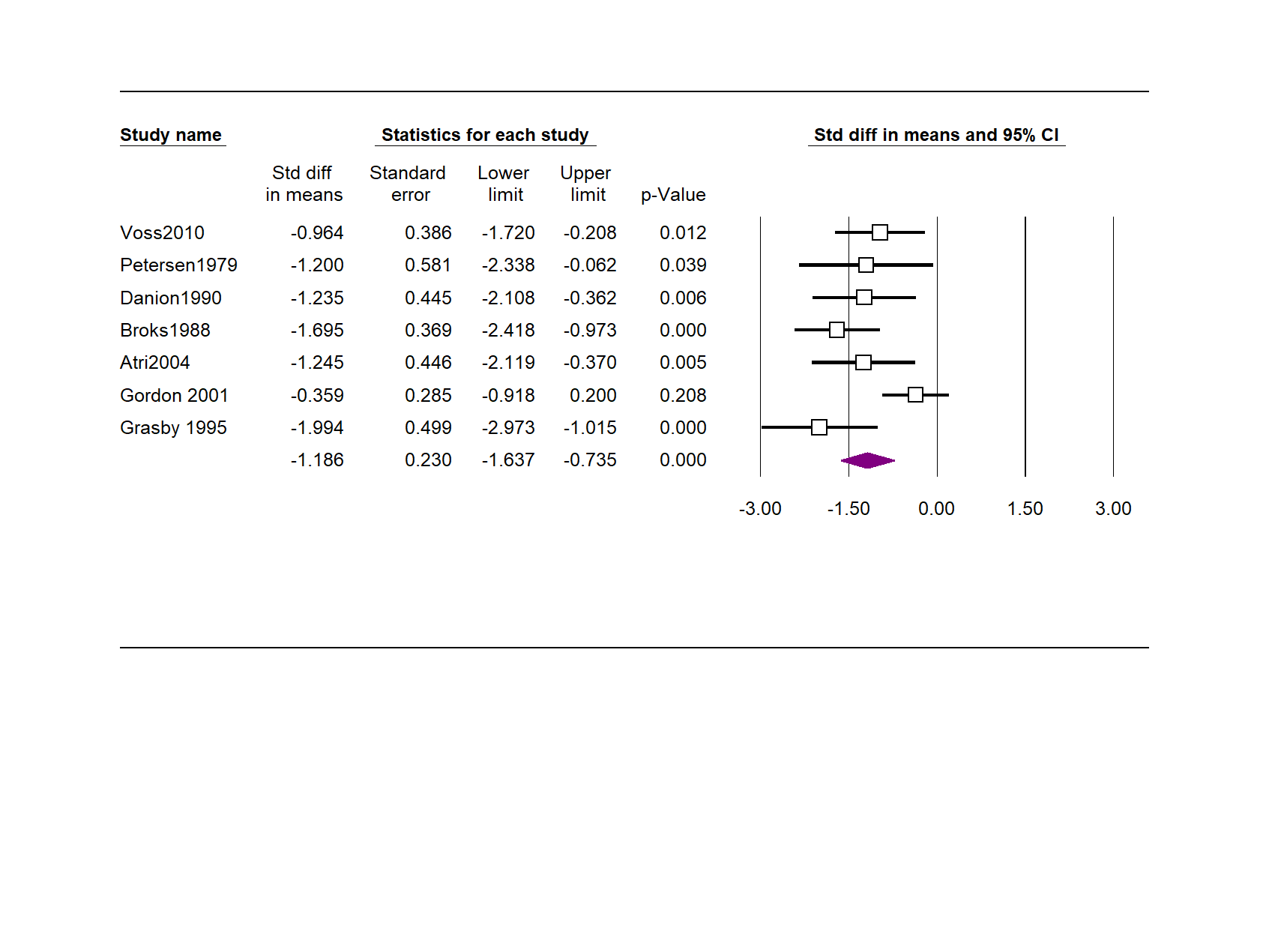


Forest plot of assessment of effects of scopolamine on free recall.


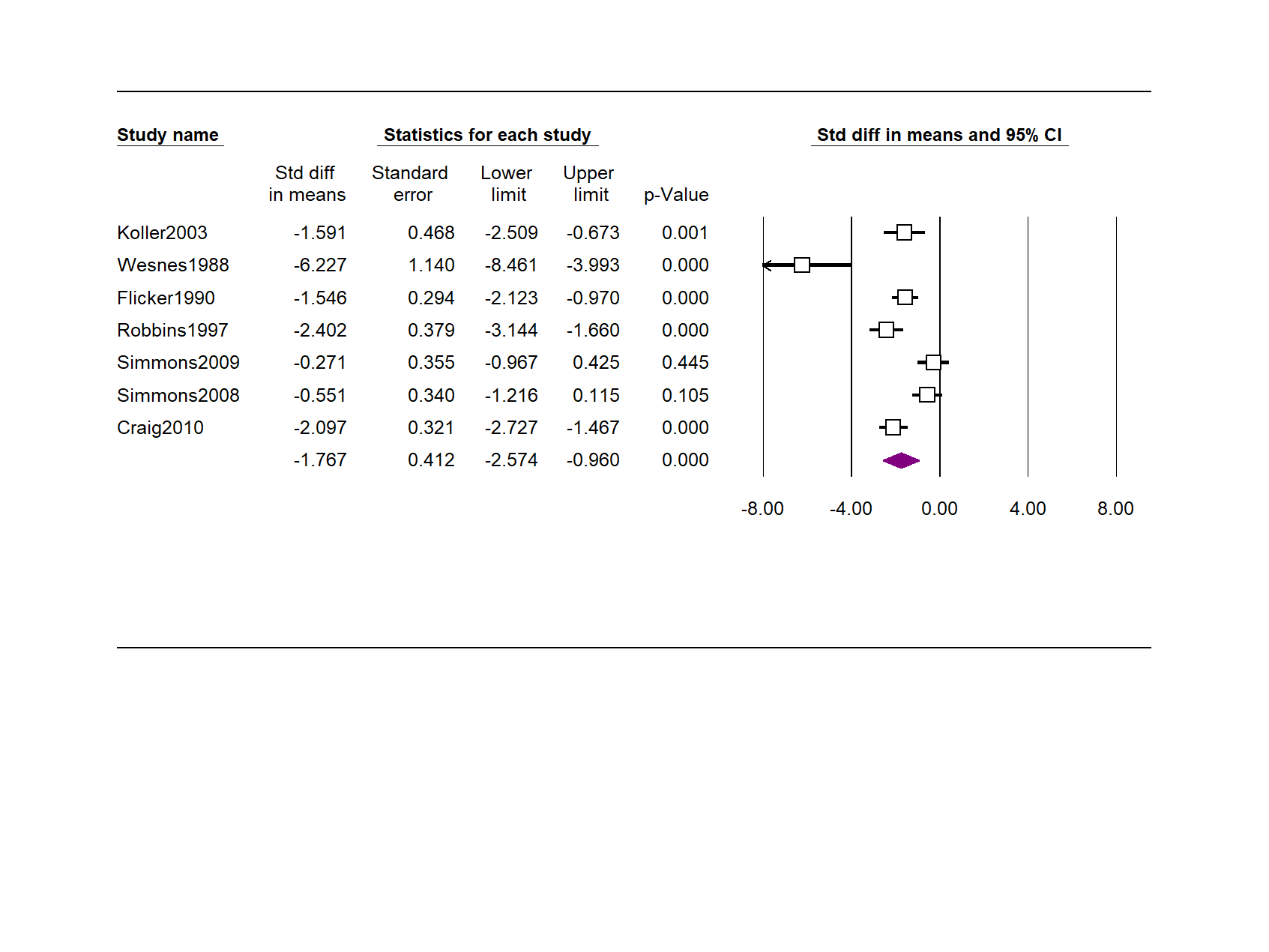


Forest plot of assessment of effects of scopolamine on matching to sample.


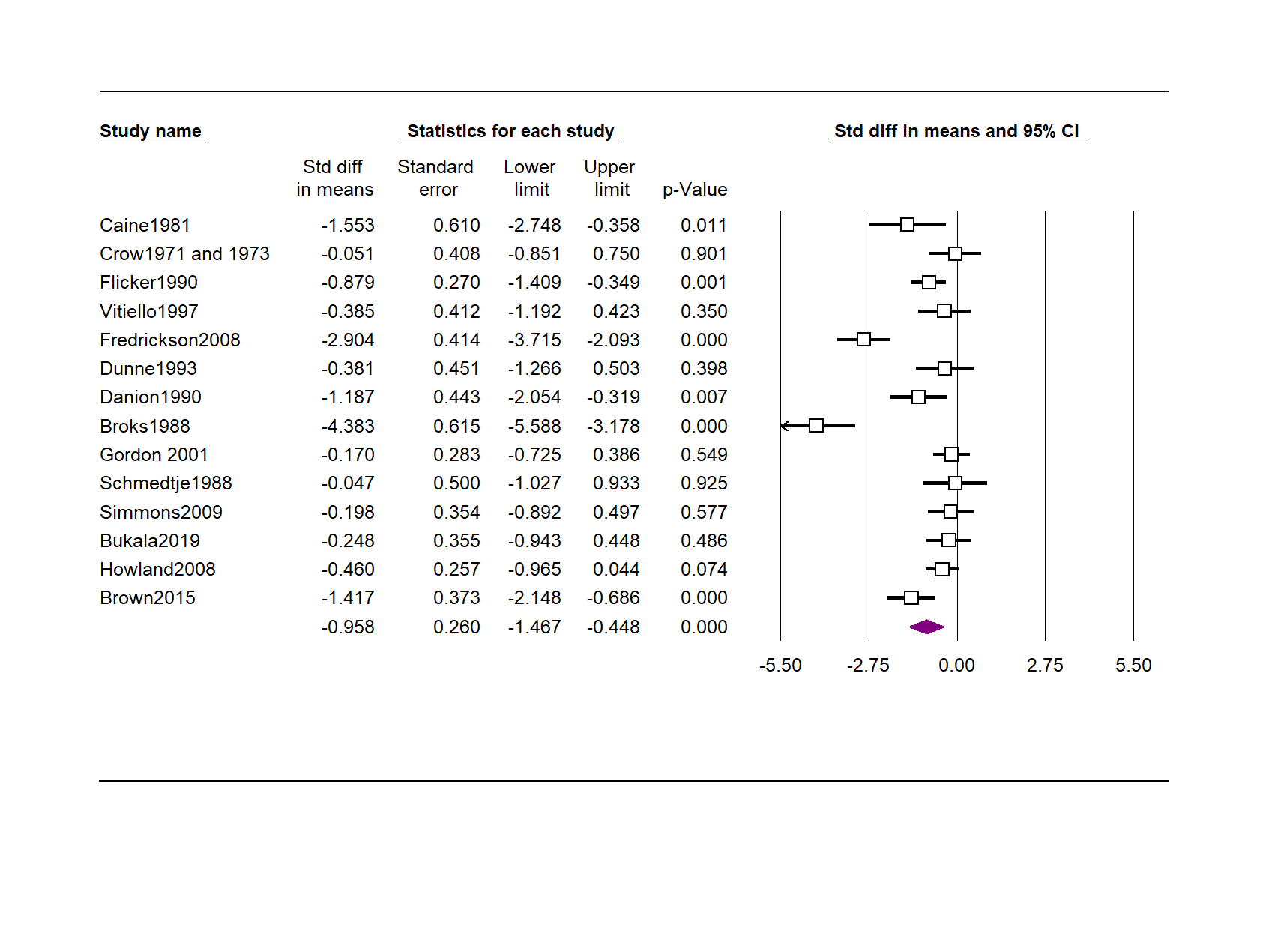


Forest plot of assessment of effects of scopolamine on complex cognitive tasks.


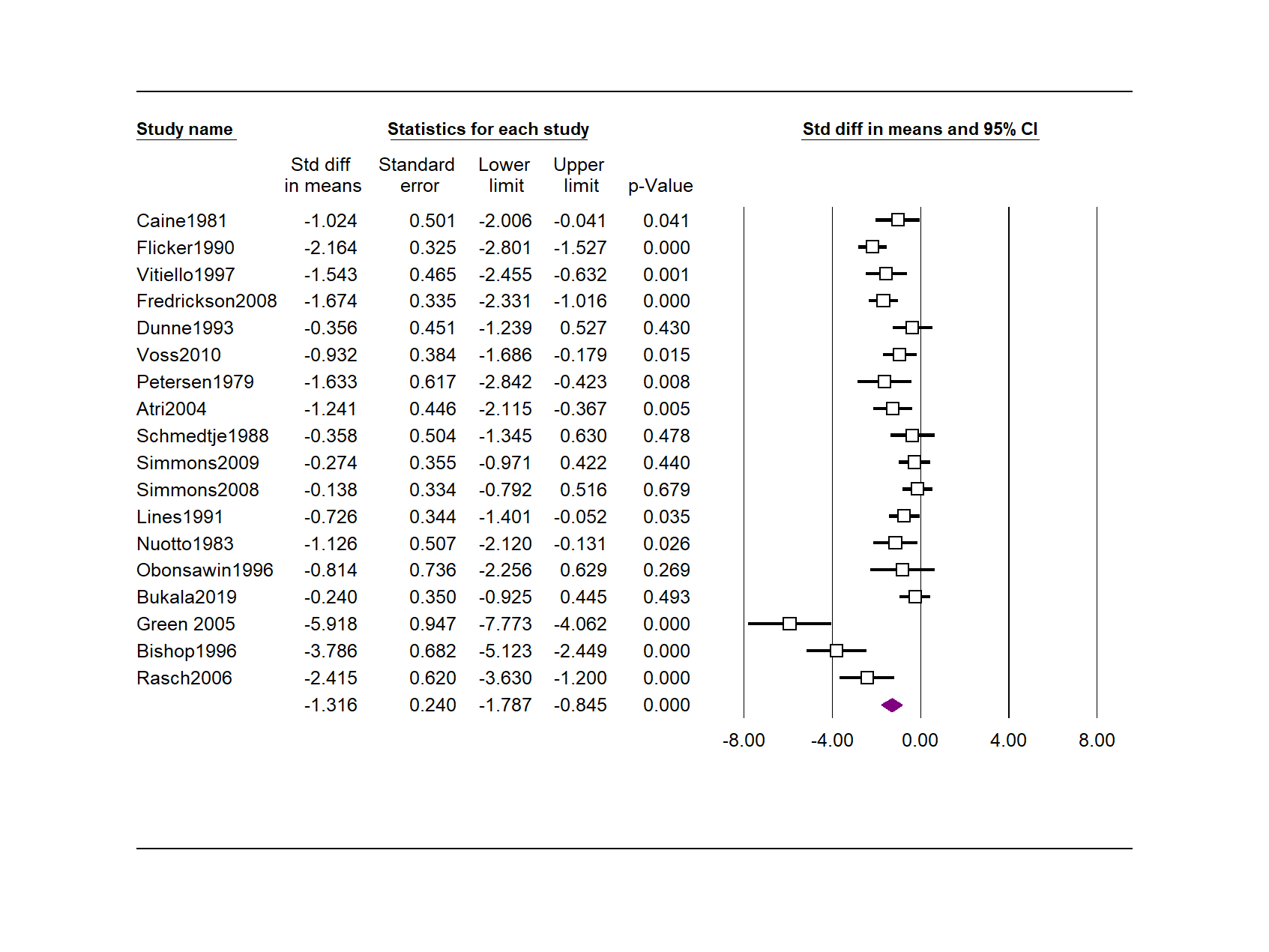


Forest plot of assessment of effects of scopolamine on complex memory tasks.


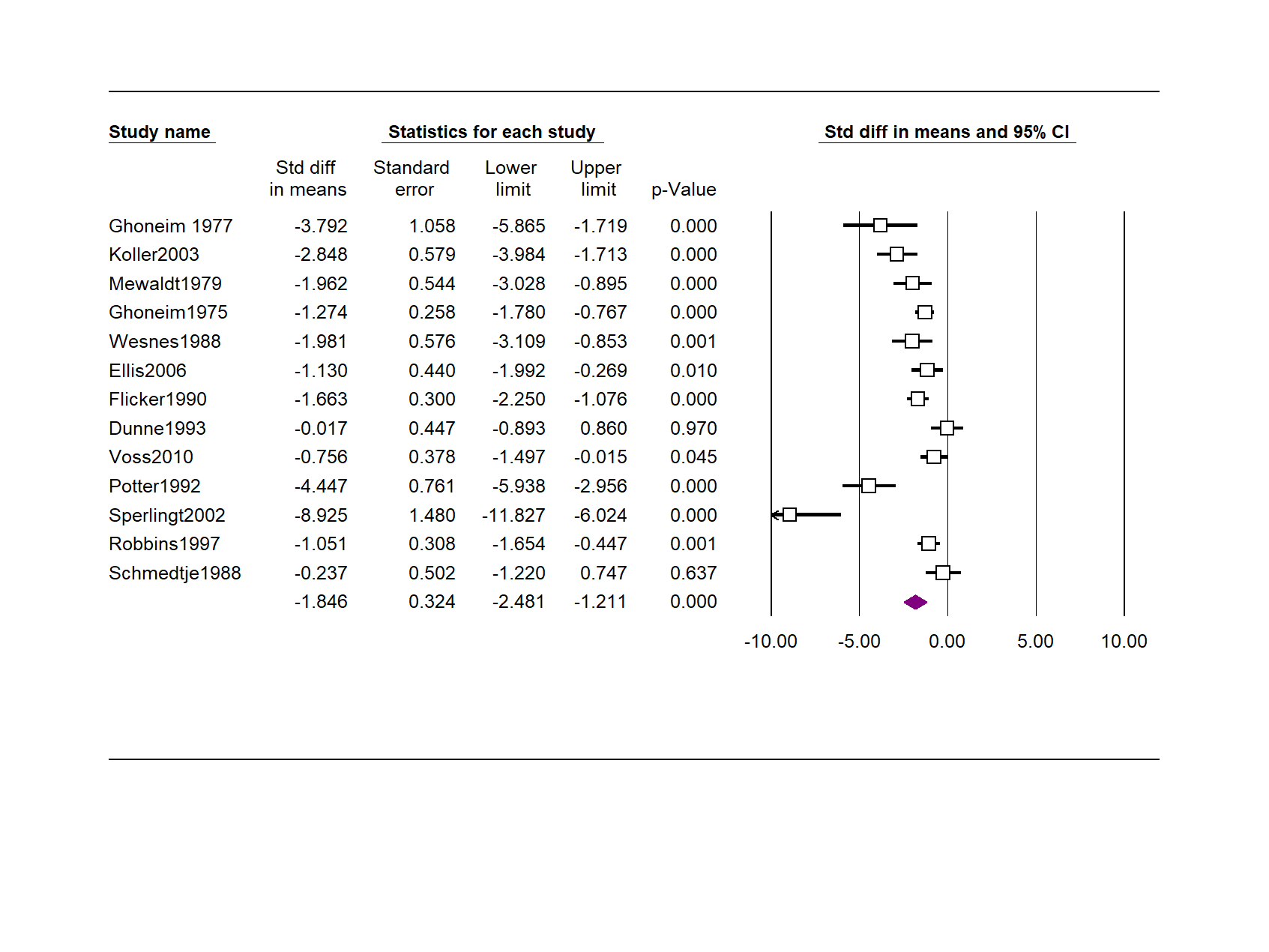


Forest plot of assessment of effects of scopolamine on recognition.
