# Supplementary material for: Cognitive effects of individual anticholinergic drugs: a systematic review and meta-analysis
Source: Dement Neuropsychol. 2023 May 29;17:e20220053. doi: 10.1590/1980-5764-DN-2022-0053 (PMC10229087; doi:10.1590/1980-5764-DN-2022-0053)
Supplement: Supplementary file 4 [file 1980-5764-DN-17-e20220053-Suppl04.docx]

**Supplementary Material 4**

| **RANDOMIZED CONTROLLED TRIALS** | | | | | | | | | | | | | | | | | | | | | |
| --- | --- | --- | --- | --- | --- | --- | --- | --- | --- | --- | --- | --- | --- | --- | --- | --- | --- | --- | --- | --- | --- |
| **Study** | **Q1** | **Q2** | | **Q3** | | **Q4** | **Q5** | | **Q6** | | **Q7** | **Q8** | | **Q9** | | **Q10** | **Q11** | | **Q12** | | **Q13** |
| **Bishop 1996** | Unclear | Unclear | | Unclear | | Yes | Yes | | Yes | | Yes | Yes | | Yes | | Yes | Yes | | Yes | | Yes |
| **Brown 2015** | Unclear | Unclear | | Unclear | | Yes | Yes | | Yes | | Yes | Yes | | Yes | | Yes | Yes | | Yes | | Yes |
| **Brown 2016** | Unclear | Unclear | | Unclear | | Yes | Yes | | Yes | | Yes | Yes | | Yes | | Yes | Yes | | Yes | | Yes |
| **Bukala 2019** | Unclear | Unclear | | Yes | | Yes | Yes | | Yes | | Yes | Yes | | Yes | | Yes | Yes | | Yes | | Yes |
| **Caine 1981** | Unclear | Unclear | | Unclear | | No | Unclear | | Unclear | | Unclear | Yes | | Yes | | Yes | Yes | | Yes | | Yes |
| **Callaway 1985** | Unclear | Unclear | | Unclear | | Yes | Yes | | Yes | | Yes | Yes | | Yes | | Yes | Yes | | Yes | | Yes |
| **Chhatwal 2018** | Unclear | Unclear | | Unclear | | Yes | Yes | | Yes | | Yes | Yes | | Yes | | Yes | Yes | | Yes | | Yes |
| **Curran 1991** | Unclear | Unclear | | Yes | | Yes | Yes | | Yes | | Yes | Yes | | Yes | | Yes | Yes | | Yes | | Yes |
| **Danion 1990** | Unclear | Unclear | | Unclear | | Yes | Yes | | Yes | | Yes | Yes | | Yes | | Yes | Yes | | Yes | | Yes |
| **Diefenbach 2003** | Yes | Yes | | Unclear | | Yes | Yes | | Yes | | Yes | Yes | | Yes | | Yes | Yes | | Yes | | Yes |
| **Diefenbach 2005** | Yes | Yes | | Unclear | | Yes | Yes | | Yes | | Yes | Yes | | Yes | | Yes | Yes | | Yes | | Yes |
| **Dubeau 2014** | Yes | Yes | | Yes | | Yes | Yes | | Yes | | Yes | Yes | | Yes | | Yes | Yes | | Yes | | Yes |
| **Duka 1992** | Unclear | Unclear | | Yes | | Yes | Yes | | Yes | | Yes | Yes | | Yes | | Yes | Yes | | Yes | | Yes |
| **Duka 1995 and 1996** | Unclear | Unclear | | Yes | | Yes | Yes | | Yes | | Yes | Yes | | Yes | | Yes | Yes | | Yes | | Yes |
| **Dundee 1972** | Unclear | Unclear | | Unclear | | Yes | Yes | | Yes | | Yes | Yes | | Yes | | Yes | Yes | | Yes | | Yes |
| **Ebert 1998** | Unclear | Unclear | | Unclear | | Yes | Yes | | Yes | | Yes | Yes | | Yes | | Yes | Yes | | Yes | | Yes |
| **Edginton 2003** | Unclear | Unclear | | Unclear | | Yes | Yes | | Yes | | Yes | Yes | | Yes | | Yes | Yes | | Yes | | Yes |
| **Ellis 2006** | Unclear | Unclear | | Yes | | Yes | Yes | | Yes | | Yes | Yes | | Yes | | Yes | Yes | | Yes | | Yes |
| **Erskine 2004** | Unclear | Unclear | | Yes | | Yes | Yes | | Yes | | Yes | Yes | | Yes | | Yes | Yes | | Yes | | Yes |
| **Fredrickson 2008** | Yes | Yes | | Yes | | Yes | Yes | | Yes | | Yes | Yes | | Yes | | Yes | Yes | | Yes | | Yes |
| **Frith 1989** | Unclear | Unclear | | Yes | | Unclear | Unclear | | Unclear | | Yes | Yes | | Yes | | Yes | Yes | | Yes | | Yes |
| **Geller 2017** | Yes | Yes | | Yes | | Yes | Yes | | Yes | | Yes | Yes | | Yes | | Yes | Yes | | Yes | | Yes |
| **Giramonti 2008** | Unclear | Unclear | | Yes | | Yes | Yes | | Yes | | Yes | Yes | | Yes | | Yes | Yes | | Yes | | Yes |
| **Golding 2018** | Unclear | Unclear | | Unclear | | Yes | Yes | | Yes | | Yes | Yes | | Yes | | Yes | Yes | | Yes | | Yes |
| **Gordon 2001** | Unclear | Unclear | | Unclear | | Yes | Yes | | Yes | | Yes | Yes | | Yes | | Yes | Yes | | Yes | | Yes |
| **Green 2005** | Yes | Yes | | Yes | | Yes | Yes | | Yes | | Yes | Yes | | Yes | | Yes | Yes | | Yes | | Yes |
| **Grober 1989** | Unclear | Unclear | | Yes | | Yes | Yes | | Yes | | Yes | Yes | | Yes | | Yes | Yes | | Yes | | Yes |
| **Harel 2013** | Unclear | Unclear | | Unclear | | Yes | Yes | | Yes | | Yes | Yes | | Yes | | Yes | Yes | | Yes | | Yes |
| **Hongyu 2019** | Yes | Yes | | Yes | | Unclear | Unclear | | Unclear | | Yes | Yes | | Yes | | Yes | Yes | | Yes | | Yes |
| **Howland 2008** | Unclear | Unclear | | Unclear | | Yes | Yes | | Yes | | Yes | Yes | | Yes | | Yes | Yes | | Yes | | Yes |
| **Jones 1979** | Unclear | Unclear | | Unclear | | Yes | Yes | | Yes | | Yes | Yes | | Yes | | Yes | Yes | | Yes | | Yes |
| **Kamboj 2006a** | Unclear | Unclear | | Yes | | Yes | Yes | | Yes | | Yes | Yes | | Yes | | Yes | Yes | | Yes | | Yes |
| **Kamboj 2006b** | Unclear | Unclear | | Yes | | Yes | Yes | | Yes | | Yes | Yes | | Yes | | Yes | Yes | | Yes | | Yes |
| **Katz 1998** | Unclear | Unclear | | Yes | | Yes | Yes | | Yes | | Yes | Yes | | Yes | | Yes | Yes | | Yes | | Yes |
| **Kay 2005** | Yes | Yes | | Unclear | | Yes | Yes | | Yes | | Yes | Yes | | Yes | | Yes | Yes | | Yes | | Yes |
| **Kay 2006** | Unclear | Unclear | | Yes | | Yes | Yes | | Yes | | Yes | Yes | | Yes | | Yes | Yes | | Yes | | Yes |
| **Kay 2012a** | Unclear | Unclear | | Yes | | Yes | Yes | | Yes | | Yes | Yes | | Yes | | Yes | Yes | | Yes | | Yes |
| **Kay 2012b** | Yes | Yes | | Yes | | Yes | Yes | | Yes | | Yes | Yes | | Yes | | Yes | Yes | | Yes | | Yes |
| **Koller 2003** | Yes | Yes | | Yes | | Yes | Yes | | Yes | | Yes | Yes | | Yes | | Yes | Yes | | Yes | | Yes |
| **Kosilov 2018** | Unclear | Unclear | | Yes | | No | No | | No | | Yes | Yes | | Yes | | Yes | Yes | | Yes | | Yes |
| **Lavoie 2019** | Unclear | Unclear | | Yes | | Yes | Yes | | Yes | | Yes | Yes | | Yes | | Yes | Yes | | Yes | | Yes |
| **Lipton 2005** | Yes | Yes | | Yes | | Yes | Yes | | Yes | | Yes | Yes | | Yes | | Yes | Yes | | Yes | | Yes |
| **Little 1995** | Unclear | Unclear | | Yes | | Yes | Yes | | Yes | | Yes | Yes | | Yes | | Yes | Yes | | Yes | | Yes |
| **Little 1998** | Unclear | Unclear | | Unclear | | Yes | Yes | | Yes | | Yes | Yes | | Yes | | Yes | Yes | | Yes | | Yes |
| **Mewaldt 1979** | Unclear | Unclear | | Yes | | Yes | Yes | | Yes | | Yes | Yes | | Yes | | Yes | Yes | | Yes | | Yes |
| **Mintzer 2001** | Unclear | Unclear | | Yes | | Yes | Yes | | Yes | | Yes | Yes | | Yes | | Yes | Yes | | Yes | | Yes |
| **Mintzer 2003** | Unclear | Unclear | | Unclear | | Yes | Yes | | Yes | | Yes | Yes | | Yes | | Yes | Yes | | Yes | | Yes |
| **Mintzer 2005** | Unclear | Unclear | | Yes | | Yes | Yes | | Yes | | Yes | Yes | | Yes | | Yes | Yes | | Yes | | Yes |
| **Mintzer 2007** | Unclear | Unclear | | Yes | | Yes | Yes | | Yes | | Yes | Yes | | Yes | | Yes | Yes | | Yes | | Yes |
| **Mintzer 2010** | Unclear | Unclear | | Unclear | | Yes | Yes | | Yes | | Yes | Yes | | Yes | | Yes | Yes | | Yes | | Yes |
| **Nakra 1992** | Unclear | Unclear | | Unclear | | Yes | Yes | | Yes | | Yes | Yes | | Yes | | Yes | Yes | | Yes | | Yes |
| **Nuotto 1983** | Yes | Yes | | Yes | | Yes | Yes | | Yes | | Yes | Yes | | Yes | | Yes | Yes | | Yes | | Yes |
| **Petersen 1977** | Unclear | Unclear | | Unclear | | No | No | | No | | Yes | Yes | | Yes | | Yes | Yes | | Yes | | Yes |
| **Petersen 1979** | Unclear | Unclear | | Unclear | | No | No | | No | | Yes | Yes | | Yes | | Yes | Yes | | Yes | | Yes |
| **Pomara 2004** | Unclear | Unclear | | Yes | | Yes | Yes | | Yes | | Yes | Yes | | Yes | | Yes | Yes | | Yes | | Yes |
| **Pomara 2008** | Unclear | Unclear | | Yes | | Yes | Yes | | Yes | | Yes | Yes | | Yes | | Yes | Yes | | Yes | | Yes |
| **Rabey 1996** | Unclear | Unclear | | Unclear | | Yes | Yes | | Yes | | Yes | Yes | | Yes | | Yes | Yes | | Yes | | Yes |
| **Ramsdell 1996** | Unclear | Unclear | | Yes | | Yes | Yes | | Yes | | Yes | Yes | | Yes | | Yes | Yes | | Yes | | Yes |
| **Richardson 1984** | Unclear | Unclear | | Yes | | Yes | Yes | | Yes | | Yes | Yes | | Yes | | Yes | Yes | | Yes | | Yes |
| **Richardson 1985** | Unclear | Unclear | | Unclear | | Yes | Yes | | Yes | | Yes | Yes | | Yes | | Yes | Yes | | Yes | | Yes |
| **Roh 2013** | Yes | Yes | | Yes | | Yes | Yes | | Yes | | Yes | Yes | | Yes | | Yes | Yes | | Yes | | Yes |
| **Rosier 1998** | Unclear | Unclear | | Unclear | | No | No | | No | | Yes | Yes | | Yes | | Yes | Yes | | Yes | | Yes |
| **Rusted 1991** | Unclear | Unclear | | Yes | | Yes | Yes | | Yes | | Yes | Yes | | Yes | | Yes | Yes | | Yes | | Yes |
| **Schifano 1994** | Unclear | Unclear | | Yes | | Yes | Yes | | Yes | | Yes | Yes | | Yes | | Yes | Yes | | Yes | | Yes |
| **Schmedtje 1988** | Unclear | Unclear | | Yes | | Yes | Yes | | Yes | | Yes | Yes | | Yes | | Yes | Yes | | Yes | | Yes |
| **Simmons 2008** | Unclear | Unclear | | Yes | | Yes | Yes | | Yes | | Yes | Yes | | Yes | | Yes | Yes | | Yes | | Yes |
| **Simmons 2009** | Unclear | Unclear | | Yes | | Yes | Yes | | Yes | | Yes | Yes | | Yes | | Yes | Yes | | Yes | | Yes |
| **Simmons 2010** | Unclear | Unclear | | Yes | | Yes | Yes | | Yes | | Yes | Yes | | Yes | | Yes | Yes | | Yes | | Yes |
| **Sperling 2002** | Unclear | Unclear | | Yes | | Yes | Yes | | Yes | | Yes | Yes | | Yes | | Yes | Yes | | Yes | | Yes |
| **Sunderland 1987** | Unclear | Unclear | | Unclear | | Yes | Yes | | Yes | | Yes | Yes | | Yes | | Yes | Yes | | Yes | | Yes |
| **Tariot 1996** | Unclear | Unclear | | Yes | | Yes | Yes | | Yes | | Yes | Yes | | Yes | | Yes | Yes | | Yes | | Yes |
| **Vitiello 1997** | Unclear | Unclear | | Yes | | Yes | Yes | | Yes | | Yes | Yes | | Yes | | Yes | Yes | | Yes | | Yes |
| **Wagg 2013** | Yes | Yes | | Yes | | Yes | Yes | | Yes | | Yes | Yes | | Yes | | Yes | Yes | | Yes | | Yes |
| **Wesnes 2009** | Unclear | Unclear | | Unclear | | Yes | Yes | | Yes | | Yes | Yes | | Yes | | Yes | Yes | | Yes | | Yes |
| **Wetherell 1980** | Yes | Yes | | Yes | | Yes | Yes | | Yes | | Yes | Yes | | Yes | | Yes | Yes | | Yes | | Yes |
| **QUASI-EXPERIMENTAL STUDIES** | | | | | | | | | | | | | | | | | | | | | |
| **Study** | **Q1** | | **Q2** | | **Q3** | | | **Q4** | | **Q5** | | | **Q6** | | **Q7** | | | **Q8** | | **Q9** | |
| **Atri 2004** | Yes | | Unclear | | Yes | | | Yes | | Yes | | | Yes | | Yes | | | Yes | | Yes | |
| **Baakman 2017** | Yes | | Unclear | | Yes | | | Yes | | Yes | | | Yes | | Yes | | | Yes | | Yes | |
| **Beatty 1986** | Yes | | Unclear | | Yes | | | Yes | | No | | | Yes | | Yes | | | Yes | | Yes | |
| **Borghans 2017** | Yes | | Unclear | | Yes | | | Yes | | No | | | Yes | | Yes | | | Yes | | Yes | |
| **Borghans 2020** | Yes | | Unclear | | Yes | | | Yes | | Yes | | | Yes | | Yes | | | Yes | | Yes | |
| **Brandeis 1992** | Yes | | Unclear | | Yes | | | Yes | | Yes | | | Yes | | Yes | | | Yes | | Yes | |
| **Broks 1988** | Yes | | Unclear | | Yes | | | Yes | | Yes | | | Yes | | Yes | | | Yes | | Yes | |
| **Callaway 1958** | Yes | | Unclear | | Yes | | | Yes | | No | | | Yes | | Yes | | | Yes | | Yes | |
| **Craig 2010** | Yes | | Yes | | Yes | | | Yes | | Yes | | | Yes | | Yes | | | Yes | | Yes | |
| **Crow 1971 and 1973** | Yes | | Unclear | | Unclear | | | Yes | | No | | | Yes | | Yes | | | Yes | | Yes | |
| **Crow 1975** | Yes | | Unclear | | Unclear | | | Yes | | No | | | Yes | | Yes | | | Yes | | Yes | |
| **Drachman 1974** | Yes | | Unclear | | Yes | | | Yes | | No | | | Yes | | Yes | | | Yes | | Yes | |
| **Dunne 1985** | Yes | | Unclear | | Yes | | | Yes | | No | | | Yes | | Yes | | | Yes | | Yes | |
| **Dunne 1986** | Yes | | Unclear | | Yes | | | Yes | | Yes | | | Yes | | Yes | | | Yes | | Yes | |
| **Dunne 1990** | Yes | | Unclear | | Yes | | | Yes | | No | | | Yes | | Yes | | | Yes | | Yes | |
| **Dunne 1993** | Yes | | Unclear | | Yes | | | Yes | | No | | | Yes | | Yes | | | Yes | | Yes | |
| **Flicker 1990** | Yes | | No | | Yes | | | Yes | | No | | | Yes | | Yes | | | Yes | | Yes | |
| **Flicker 1992** | Yes | | No | | Yes | | | Yes | | No | | | Yes | | Yes | | | Yes | | Yes | |
| **Frith 1984** | Yes | | Unclear | | Yes | | | Yes | | No | | | Yes | | Yes | | | Yes | | Yes | |
| **Ghoneim 1975** | Yes | | Unclear | | Yes | | | Yes | | No | | | Yes | | Yes | | | Yes | | Yes | |
| **Ghoneim 1977** | Yes | | Unclear | | Yes | | | Yes | | No | | | Yes | | Yes | | | Yes | | Yes | |
| **Golding 1989** | Yes | | Yes | | Yes | | | Yes | | Yes | | | Yes | | Yes | | | Yes | | Yes | |
| **Grasby 1995** | Yes | | Unclear | | Yes | | | Yes | | Yes | | | Yes | | Yes | | | Yes | | Yes | |
| **Hardy 1962** | Yes | | Unclear | | Yes | | | Yes | | No | | | Yes | | Yes | | | Yes | | Yes | |
| **Higgins 1989** | Yes | | Yes | | Yes | | | Yes | | Yes | | | Yes | | Yes | | | Yes | | Yes | |
| **Jacob Huff 1988** | Yes | | Yes | | Yes | | | Yes | | No | | | Yes | | Yes | | | Yes | | Yes | |
| **Kamboj 2006 a** | Yes | | Unclear | | Yes | | | Yes | | Yes | | | Yes | | Yes | | | Yes | | Yes | |
| **Kamboj 2006 b** | Yes | | Unclear | | Yes | | | Yes | | Yes | | | Yes | | Yes | | | Yes | | Yes | |
| **Katz 1998** | Yes | | Yes | | Yes | | | Yes | | Yes | | | Yes | | Yes | | | Yes | | Yes | |
| **Kay 2005** | Yes | | Yes | | Yes | | | Yes | | Yes | | | Yes | | Yes | | | Yes | | Yes | |
| **Klinkenberg 2012** | Yes | | Yes | | Yes | | | Yes | | Yes | | | Yes | | Yes | | | Yes | | Yes | |
| **Liem-moolenaar 2011** | Yes | | Yes | | Yes | | | Yes | | Yes | | | Yes | | Yes | | | Yes | | Yes | |
| **Lines 1991** | Yes | | Unclear | | Yes | | | Yes | | No | | | Yes | | Yes | | | Yes | | Yes | |
| **Newhouse 1992** | Yes | | Unclear | | Yes | | | Yes | | Yes | | | Yes | | Yes | | | Yes | | Yes | |
| **Newhouse 1994** | Yes | | Unclear | | Yes | | | Yes | | Yes | | | Yes | | Yes | | | Yes | | Yes | |
| **Obonsawin 1996** | Yes | | Unclear | | Yes | | | Yes | | No | | | Yes | | Yes | | | Yes | | Yes | |
| **Parrott 1985** | Yes | | Yes | | Yes | | | Yes | | Yes | | | Yes | | Yes | | | Yes | | Yes | |
| **Parrott 1986** | Yes | | Yes | | Yes | | | Yes | | Yes | | | Yes | | Yes | | | Yes | | Yes | |
| **Parrott 1988** | Yes | | Yes | | Yes | | | Yes | | Yes | | | Yes | | Yes | | | Yes | | Yes | |
| **Parrott 1990** | Yes | | Yes | | Yes | | | Yes | | Yes | | | Yes | | Yes | | | Yes | | Yes | |
| **Paule 2004** | Yes | | Yes | | Yes | | | Yes | | No | | | Yes | | Yes | | | Yes | | Yes | |
| **Pomara 2010** | Yes | | Yes | | Yes | | | Yes | | No | | | Yes | | Yes | | | Yes | | Yes | |
| **Pompeia 2002** | Yes | | Yes | | Yes | | | Yes | | Yes | | | Yes | | Yes | | | Yes | | Yes | |
| **Potamianos 1982** | Yes | | Unclear | | Yes | | | Yes | | No | | | Yes | | Yes | | | Yes | | Yes | |
| **Potter 1992** | Yes | | Unclear | | Yes | | | Yes | | No | | | Yes | | Yes | | | Yes | | Yes | |
| **Preston 1988** | Yes | | Yes | | Yes | | | Yes | | No | | | Yes | | Yes | | | Yes | | Yes | |
| **Rasch 2006** | Yes | | Yes | | Yes | | | Yes | | No | | | Yes | | Yes | | | Yes | | Yes | |
| **Rasmusson 1979** | Yes | | Unclear | | Yes | | | Yes | | No | | | Yes | | Yes | | | Yes | | Yes | |
| **Robbins 1997** | Yes | | Unclear | | Yes | | | Yes | | Yes | | | Yes | | Yes | | | Yes | | Yes | |
| **Rusted 1988a** | Yes | | Unclear | | Yes | | | Yes | | Yes | | | Yes | | Yes | | | Yes | | Yes | |
| **Rusted 1988a** | Yes | | Unclear | | Yes | | | Yes | | Yes | | | Yes | | Yes | | | Yes | | Yes | |
| **Rusted 1989** | Yes | | Unclear | | Yes | | | Yes | | Yes | | | Yes | | Yes | | | Yes | | Yes | |
| **Sambeth 2015** | Yes | | Yes | | Yes | | | Yes | | Yes | | | Yes | | Yes | | | Yes | | Yes | |
| **Sherman 2003** | Yes | | Unclear | | Yes | | | Yes | | No | | | Yes | | Yes | | | Yes | | Yes | |
| **Sommer 2005** | Yes | | Unclear | | Yes | | | Yes | | No | | | Yes | | Yes | | | Yes | | Yes | |
| **Staskin 2010** | Yes | | Unclear | | Unclear | | | Yes | | No | | | Yes | | Yes | | | Yes | | Yes | |
| **Thompson 2000** | Yes | | Yes | | Yes | | | Yes | | No | | | Yes | | Yes | | | Yes | | Yes | |
| **Tröster 1989** | Yes | | Yes | | Yes | | | Yes | | Yes | | | Yes | | Yes | | | Yes | | Yes | |
| **Voss 2010** | Yes | | Yes | | Yes | | | Yes | | No | | | Yes | | Yes | | | Yes | | Yes | |
| **Wesnes 1983** | Yes | | Unclear | | Yes | | | Yes | | No | | | Yes | | Yes | | | Yes | | Yes | |
| **Wesnes 1984a** | Yes | | Unclear | | Yes | | | Yes | | No | | | Yes | | Yes | | | Yes | | Yes | |
| **Wesnes 1984b** | Yes | | Unclear | | Yes | | | Yes | | No | | | Yes | | Yes | | | Yes | | Yes | |
| **Wesnes 1988** | Yes | | Unclear | | Yes | | | Yes | | No | | | Yes | | Yes | | | Yes | | Yes | |

Results of risk of bias assessment

Risk of Bias in Randomized Controlled Trials.

Risk of Bias in Quasi Experimental Trials.

Funnel plot of the studies after removing one different one.

.
